# Dynamic Hemispheric Lateralization of Naturalistic Emotional Processing After Unilateral Stroke: An EEG Study

**DOI:** 10.1002/cns.71096

**Published:** 2026-08-20

**Authors:** Menglin Han, Yuting Tang, Yunyi Lv, Xiaoyun Chen, Xiaocao Liu, Zhuoming Chen, Jinyi Long

**Affiliations:** ^1^ Department of Rehabilitation Medicine The First Affiliated Hospital of Jinan University Guangzhou China; ^2^ Lab of Regenerative Medicine in Sports Science, School of Physical Education and Sports Science South China Normal University Guangzhou China; ^3^ College of Information Science and Technology Jinan University Guangzhou China

**Keywords:** electroencephalography, emotion, hemisphere lesion, neural dynamics, stroke

## Abstract

**Aim:**

Emotional impairment is common after stroke, but the neural electrophysiological mechanisms by which left‐ and right‐hemisphere lesions differentially alter emotional processing remain unclear. Conventional time‐averaged measures may overlook its dynamic features in the temporal organization of emotional processing. This study aimed to characterize hemisphere‐group‐associated differences in dynamic hemispheric lateralization during naturalistic emotional processing after unilateral stroke.

**Methods:**

In this cross‐sectional study, 15 patients with left‐hemisphere stroke (L‐stroke), 15 patients with right‐hemisphere stroke (R‐stroke), and 15 healthy controls viewed validated positive, neutral, and negative film clips during 64‐channel electroencephalography recording. Self‐Assessment Manikin ratings were collected after each clip. We computed spectral power, emotion‐averaged lateralization index (LI), inter‐clip LI stability, and within‐clip LI dynamics (detectability, switching rate, and entropy). Mixed‐effects models accounted for repeated observations within participants and, where applicable, variability across film clips.

**Results:**

Stroke patients showed reduced valence and arousal responses to positive clips, particularly in R‐stroke patients. Low‐frequency spectral power analysis showed preserved emotion modulation, whereas emotion‐averaged LI mainly reflected group‐dependent hemispheric bias. Within‐clip parietal‐theta LI dynamics revealed hemispheric differences: L‐stroke showed higher detectability and lower switching, while R‐stroke showed increased switching and entropy during positive clips. Switching showed a group‐dependent association with arousal in the primary model, with stronger coupling in R‐stroke.

**Conclusion:**

Dynamic parietal‐theta lateralization revealed hemisphere‐group‐associated differences in the stability–flexibility balance of emotional brain states that were not captured by time‐averaged measures. These findings suggest that parietal‐theta LI dynamics may provide a preliminary candidate electrophysiological feature for characterizing post‐stroke emotional dysfunction.

## Graphical abstract

Unilateral stroke differentially disrupts dynamic hemispheric lateralization during naturalistic emotional viewing. Left‐hemisphere stroke showed persistent parietal‐theta states, whereas right‐hemisphere stroke showed positive‐context variability, stronger arousal–switching coupling, and blunted positive affect. These preliminary EEG features may characterize post‐stroke emotional processing.

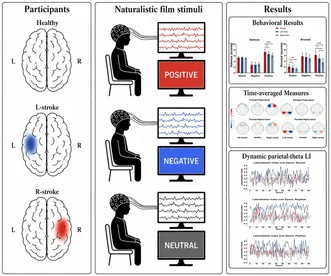

## 1 Introduction

Emotional impairments after stroke, such as depression, anxiety, or affective apathy, often persist into the chronic phase, significantly hindering functional recovery and reducing quality of life [1, 2, 3]. Despite their prevalence in clinical practice, their management remains challenging due to the unclear relationship between hemispheric lesions and emotional impairments, as well as the lack of reliable neurophysiological characteristics. Therefore, clarifying how lesions in different hemispheres affect emotional processing may improve our understanding of post‐stroke emotional impairments and help guide targeted interventions.

Current research on hemispheric lateralization has been informed by theoretical models such as the “valence hypothesis” (left‐positive/right‐negative) [4] and the “right‐hemisphere hypothesis” (global right dominance) [5]. Although these models provide useful insights, evidence from stroke cohorts regarding whether lesion laterality reliably maps onto specific affective outcomes is inconsistent [6, 7, 8]. For instance, a systematic review published in the *Lancet* found no evidence to support the hypothesis that the risk of post‐stroke depression is linked to lesion laterality [6]. The reasons for such inconsistencies remain unclear, but they may partly reflect the predominance of time‐averaged approaches, which may inadequately account for emotional impairments after stroke.

Emotional processing is intrinsically dynamic [9], involving both response intensity and the temporal organization of interhemispheric states. Hemispheric contributions may vary as emotional experience unfolds [10]. From this perspective, hemispheric lateralization should not be understood solely as a static leftward or rightward bias, but as a dynamic process of interhemispheric functional balance [11]. In particular, emotional processing may depend on how lateralized brain states are maintained, switched, and reorganized over time during ongoing experiences [12]. However, for pragmatic reasons, emotion research has largely treated emotions as stable traits or brief states, thereby overlooking their temporal evolution [13]. This time‐averaged perspective may obscure how emotional processing is organized across hemispheres over time [14, 15]. For example, similar average responses or lateralization indices may arise from markedly different dynamic patterns, such as stable maintenance, flexible switching, or excessive lability of emotion‐related brain states. This limitation is especially relevant after unilateral stroke. By disrupting interhemispheric balance, unilateral stroke provides a natural lesion model for analyzing how left‐ and right‐hemisphere lesions differentially alter the temporal organization of emotion‐related brain states.

Although traditional event‐related potential (ERP) paradigms effectively isolate discrete time‐locked components, their brief trial structures and strict time‐locking constraints limit the exploration of emotional processing in naturalistic settings [16]. In contrast, naturalistic film stimuli, owing to their rich content, can effectively elicit authentic emotional states [17], enhancing higher‐order neural processes and providing extended data segments [18] for characterizing the temporal organization of brain states [19, 20]. Evidence from healthy populations supports the importance of this dynamic perspective. Microstate analyses have shown that emotional states and regulation strategies influence the temporal dynamics of spontaneous brain activity, revealing effects that are difficult to capture through traditional static analysis [21, 22]. Under naturalistic viewing, dynamic functional connectivity has been linked to temporal fluctuations in emotional experiences [23], and its associations with subjective arousal appear more consistent than those with valence [24]. Converging evidence further implicates frontal and parietal regions in these dynamics [25, 26], particularly posterior parietal‐theta activity and frontoparietal‐theta coupling, which are thought to support integrative control within frontoparietal networks [27, 28]. However, these findings are primarily based on healthy populations, and there is a lack of temporally resolved measures of hemispheric lateralization capable of capturing this dynamic imbalance.

This study used a naturalistic viewing approach to record electroencephalography (EEG) data from individuals with left‐hemisphere stroke (L‐stroke), right‐hemisphere stroke (R‐stroke), and healthy controls (HC). The main analysis examined parietal‐theta within‐clip lateralization index (LI) dynamics, while spectral power, emotion‐averaged LI, and inter‐clip LI stability were secondary analyses. We hypothesized that the side of the lesion would differently affect the stability–flexibility balance of emotion‐related lateralized states and that these dynamics would be more closely linked to subjective arousal than to valence.

## 2 Method

### 2.1 Ethics Statement

This study was approved by the Scientific Research Ethics Committee of the First Affiliated Hospital of Jinan University (KY‐2025‐332) and was conducted in accordance with the Declaration of Helsinki. All participants provided written informed consent prior to participation.

### 2.2 Participants

Participants aged 45–80 years were right‐handed and native Chinese speakers with normal or corrected‐to‐normal vision. The study included three groups (*n* = 15 per group): Healthy older adults, patients with L‐stroke, and patients with R‐stroke.

Inclusion criteria for stroke patients were: (1) diagnosed with stroke according to the criteria established at the Fourth National Cerebrovascular Disease Academic Conference in 1995; (2) diagnosis of stroke confirmed by computed tomography (CT) or MRI; (3) first‐ever unilateral stroke; (4) recovery stage (1–6 months post‐onset); and (5) sufficient cognitive capacity to understand task instructions and complete self‐reports (Montreal Cognitive Assessment (MoCA) score > 17). Baseline clinical and affective measures (Hamilton Anxiety Rating Scale (HAMA) and Hamilton Depression Rating Scale (HAMD)) were collected for descriptive purposes and sensitivity analyses.

General exclusion criteria for all participants included: (1) history of other neurological or psychiatric disorders; (2) severe aphasia, neglect, or visual field deficits preventing task comprehension or stimulus processing; (3) defect of the skull; and (4) current use of medications with substantial central nervous system effects (e.g., sedatives/benzodiazepines, antipsychotics, anticonvulsants, or other psychoactive drugs).

### 2.3 Experimental

Emotional stimuli were adapted from the SEED paradigm [29, 30] and consisted of 12 validated Chinese film clips (Tables S1 and S2), designed to evoke one of three emotional categories: Positive, neutral, or negative (four clips per category). All participants viewed the same set of film clips. Trials were presented in a pseudo‐randomized order such that clips from the same emotional category were not presented consecutively. The detailed trial procedure is shown in Supplementary Figure S1.

### 2.4 EEG Acquisition and Data Processing

EEG was recorded using a 64‐channel Ag/AgCl cap arranged according to the 10–20 system and amplified with a Neuroscan Synamps2 amplifier (Neurosoft Inc., Sterling, VA, United States).

EEG preprocessing was performed in EEGLAB (version: 2022) running in MATLAB (version: R2020b). Data were bandpass filtered at 1–50 Hz and notch filtered (48–52 Hz). The sampling rate was changed to 256 Hz. Bad channels were identified based on persistent non‐physiological noise, flat‐line activity, or poor signal quality across most of the recording. These channels were removed and subsequently reconstructed using spherical spline interpolation, with the number of removed channels limited to 5% of the total. Bad segments containing gross artifacts were removed before independent component analysis. Candidate artifactual components were first classified using ICLabel and then visually inspected based on scalp topographies, time courses, and power spectra. Components reflecting eye blinks, eye movements, scalp muscle activity, or channel noise were removed. The cleaned data were then re‐referenced to the common average reference. The data were segmented into two‐second epochs, followed by automatic artifact rejection (±100 μV). After artifact rejection, the number of retained 2‐s epochs was calculated for each participant and condition and was summarized in Supplementary Table S3.

### 2.5 Power Spectral Density and Lateralization Index

We focused on theta (4–7 Hz) and alpha (8–13 Hz) bands within two predefined regions of interest (ROIs): Frontal (F) and parietal (P). The frontal ROI included left‐hemisphere electrodes (Fp1, AF3, F5, F3, F1) and right‐hemisphere electrodes (Fp2, AF4, F6, F4, F2) [31]. The parietal ROI included left‐hemisphere electrodes (P5, P3, P1) and right‐hemisphere electrodes (P6, P4, P2) [32]. Power spectral density (PSD) was estimated using Welch's method. Band power was obtained by integrating PSD within the theta or alpha band and averaging across electrodes within each ROI and hemisphere. PSD results are reported in dB/Hz; however, all LI analyses were computed on linear band power. For each epoch, the LI(t) was computed as (L − R) / (L + *R*), where L and R denote the mean band power averaged across electrodes in the left and right hemispheres, respectively, within a given ROI. Epoch‐level LI(t) was first averaged within each clip to obtain a clip‐level LI. Clip‐level LI values were then used to derive (i) emotion‐averaged LI across the four clips within each emotion condition and (ii) inter‐clip LI stability indicators, whereas the epoch‐resolved LI(t) series were used to quantify within‐clip LI dynamics.

### 2.6 Assessment of Inter‐Clip Lateralization Stability

To quantify the stability of hemispheric lateralization across the four clips within each emotion category, we calculated: (i) inter‐clip dispersion (SDtrial), reflecting variability in LI magnitude across clips; (ii) inter‐clip detectability (Rdet), reflecting the reliability with which a lateralized state emerged at the clip level; and (iii) directional consistency (Cdir), reflecting the stability of lateralization direction independent of magnitude.

### 2.7 Quantification of Within‐Clip LI(t) Dynamics

To capture the temporal evolution of hemispheric lateralization within each clip, three within‐clip LI dynamic indicators were computed: (i) Within‐clip detectability (R_det_win_), reflecting the proportion of epochs exhibiting a detectable lateralized state; (ii) switching rate (SwitchRate), indexing state transitions independent of transient non‐detectable periods; and (iii) state complexity (Entropy), quantified as the normalized Shannon entropy of the tri‐state occupancy distribution. Detailed formulas are provided in the Supplementary Methods.

### 2.8 Statistical Analysis

Statistical analyses were performed using R (version 4.4.2). Continuous variables are reported as mean (standard deviation) or median (interquartile range) and categorical variables as numbers (percentage). Group differences at baseline were assessed using the Kruskal–Wallis H test for continuous variables, and chi‐square tests or Fisher's exact test for categorical variables. In addition, between‐stroke‐group comparisons (L‐stroke vs. R‐stroke) were performed for type of stroke using Fisher's exact test and for time post‐stroke using the Mann–Whitney U test.

The primary EEG analysis focused on within‐clip parietal‐theta LI dynamics at τ = 0.10 [33]. PSD, emotion‐averaged LI, and inter‐clip LI stability were treated as secondary analyses, whereas other ROI × band combinations, alternative thresholds, lesion‐category analyses, and clinical‐scale correlations were considered exploratory or sensitivity analyses.

SAM ratings, PSD, emotion‐averaged LI, and inter‐clip LI stability were modeled with Group, Emotion, and Group × Emotion as fixed effects and participant as a random intercept. Within‐clip LI dynamic models additionally included Clip as a crossed random intercept. Continuous outcomes were analyzed using linear mixed‐effects models, whereas detectability and switching were analyzed using binomial GLMMs. Dispersion was log‐transformed, entropy was boundary‐corrected and logit‐transformed, and fixed effects were evaluated using Type III Wald chi‐square tests. Multiplicity was controlled using the Benjamini–Hochberg false discovery rate (FDR) within prespecified analysis families, followed by Holm‐adjusted post hoc contrasts when appropriate. Brain–behavior coupling analyses focused a priori on parietal‐theta switching rate using within‐subject standardized SAM scores. Additional model specifications and sensitivity analyses are described in the Supplementary Methods. All tests were two‐sided with α = 0.05.

## 3 Results

### 3.1 Baseline Characteristics

A total of 45 participants met the inclusion criteria and were included in the final analyses, which comprised HC, L‐stroke, and R‐stroke groups, with 15 participants in each group. Baseline demographic characteristics were similar (Table 1). Broad lesion categories were balanced between the L‐stroke and R‐stroke groups (Tables S4 and S5). The L‐stroke and R‐stroke groups did not differ significantly in stroke type, time post‐stroke, Fugl‐Meyer Assessment Scale, or Modified Barthel Index, indicating comparable clinical status with respect to post‐stroke stage, motor impairment, and daily living dependence. As expected in post‐stroke populations, stroke groups exhibited higher baseline anxiety and depressive symptom severity (*p* < 0.01), and there was a marginal group difference in cognitive performance (*p* = 0.05).

**TABLE 1:** Demographic and clinical characteristics of the study participants.

Variable	Healthy	Left stroke	Right stroke	*p*‐value
Age, years, median [IQR]	56.00 [50.50, 63.50]	56.00 [51.5, 64.0]	58.0 [52.00, 65.50]	0.87^a^
Sex, *n* (%)				1.00^b^
Male	12 (80.00%)	12 (80.00%)	12 (80.00%)	
Female	3 (20.00%)	3 (20.00%)	3 (20.00%)	
Education, *n* (%)				0.97^c^
Primary school	4 (26.67%)	4 (26.67%)	3 (20.00%)	
Junior middle school	3 (20.00%)	3 (20.00%)	4 (26.67%)	
Senior middle school	5 (33.33%)	3 (20.00%)	4 (26.67%)	
University	3 (20.00%)	5 (33.33%)	4 (26.67%)	
Type of stroke, *n* (%)				1.00^b^
Hemorrhagic	—	5 (33.33%)	5 (33.33%)	
Ischemic	—	10 (66.67%)	10 (66.67%)	
Time post‐stroke, m, median [IQR]	—	1.00 [1.00, 3.00]	1.00 [1.00, 5.00]	0.65^d^
FMA total scores, median [IQR]	—	79.00 [26.50, 89.00]	73.00 [35.50, 91.00]	0.77^d^
MBI total scores, median [IQR]	—	71.00 [51.00, 82.50]	70.00 [51.50, 91.00]	0.69^d^
MoCA total scores, median [IQR]	23.00 [21.00, 26.50]	18.00 [17.50, 24.50]	21.00 [19.50, 24.00]	0.05^a^
Initial HAMD scores, median [IQR]	3.00 [1.50, 6.00]	11.00 [8.00, 13.50]	13.00 [8.50, 18.50]	**< 0.01** ^a^
Initial HAMA scores, median [IQR]	5.00 [1.50, 5.50]	7.00 [5.00, 8.50]	7.00 [6.00, 8.50]	**< 0.01** ^a^

*Note:* Data are presented as *n* (%) for categorical variables and median [IQR] for continuous variables. Bold values indicate statistical significance (*p* < 0.05).

Abbreviations: FMA = Fugl‐Meyer Assessment Scale; MBI = Modified Barthel Index; MoCA = Montreal Cognitive Assessment; HAMD = Hamilton Depression Rating Scale; HAMA = Hamilton Anxiety Rating Scale; IQR = Interquartile Range; m = months.

^a^ Analysis by Kruskal–Wallis H test.

^b^ Analysis by Fisher's exact test.

^c^ Analysis by χ2 test.

^d^ Analysis by Mann–Whitney U test.

### 3.2 Behavioral Results

**FIGURE 1:**
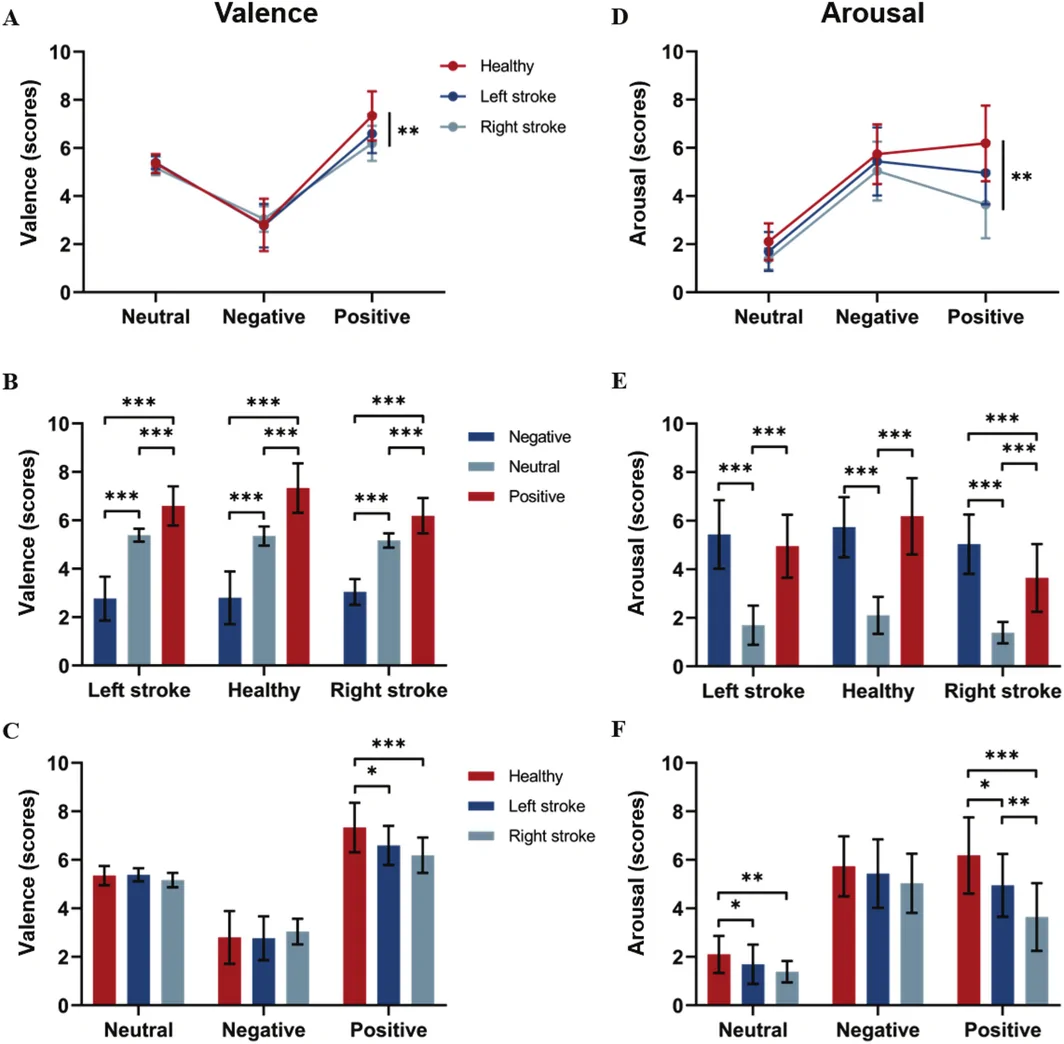
Subjective valence and arousal ratings across emotional conditions and groups. Participants rated subjective valence and arousal after each film clip using the 9‐point SAM scales (valence: 1 = very unpleasant, 9 = very pleasant; arousal: 1 = very calm, 9 = very excited). Ratings are aggregated by emotion category (negative, neutral, positive; four clips per category). (A–C) Subjective valence ratings for neutral, negative, and positive clips across three groups: Healthy controls, Left‐hemisphere stroke, and Right‐hemisphere stroke. (D–F) Subjective arousal ratings for the three emotional conditions across three groups. (A) and (D) Interaction plots illustrating the overall Group × Emotion profiles for Valence (A) and Arousal (D) ratings. (B) and (E) Within‐group comparisons (Simple effects of Emotion). Bar charts display differences in ratings between emotion conditions (Neutral, Negative, Positive) separately for Left‐stroke, Healthy, and Right‐stroke groups. (C) and (F) Between‐group comparisons (Simple effects of Group). Bar charts compare ratings across the three groups within each emotional condition. Error bars represent standard errors of the mean (SEM). *p < 0.05, **p < 0.01, ***p < 0.001.

As shown in Figure 1 and Table S6, the main effects of Emotion on both valence (χ^2^(2) = 208.460, p_FDR < 0.001) and arousal (χ^2^(2) = 174.845, p_FDR < 0.001) were significant. For valence ratings, Holm‐adjusted post hoc tests confirmed the expected within‐group ordering (negative < neutral < positive, all p_Holm < 0.001). For arousal ratings, neutral clips elicited markedly lower arousal than negative and positive clips in all groups (all p_Holm < 0.001). In sum, the emotion induction was successful.

Valence ratings revealed a significant Group × Emotion interaction (χ^2^(4) = 13.150, p_FDR = 0.013). Simple‐effects tests showed that both stroke groups reported lower positive valence than HC (L‐stroke: p_Holm = 0.028; R‐stroke: p_Holm < 0.001), with no significant group differences for neutral or negative clips. Arousal ratings also showed a significant Group (χ^2^(2) = 11.180, p_FDR = 0.006) and a Group × Emotion interaction (χ^2^(4) = 16.723, p_FDR = 0.004). For neutral and positive clips, both stroke groups reported lower arousal than HC (all p_Holm < 0.050); notably, R‐stroke exhibited significantly lower positive arousal than L‐stroke (p_Holm = 0.009). Furthermore, arousal during negative clips did not differ significantly between groups. These behavioral effects remained significant after adjustment for baseline MoCA, HAMA, and HAMD (Tables S7 and S8).

### 3.3 Power Spectral Density

**FIGURE 2:**
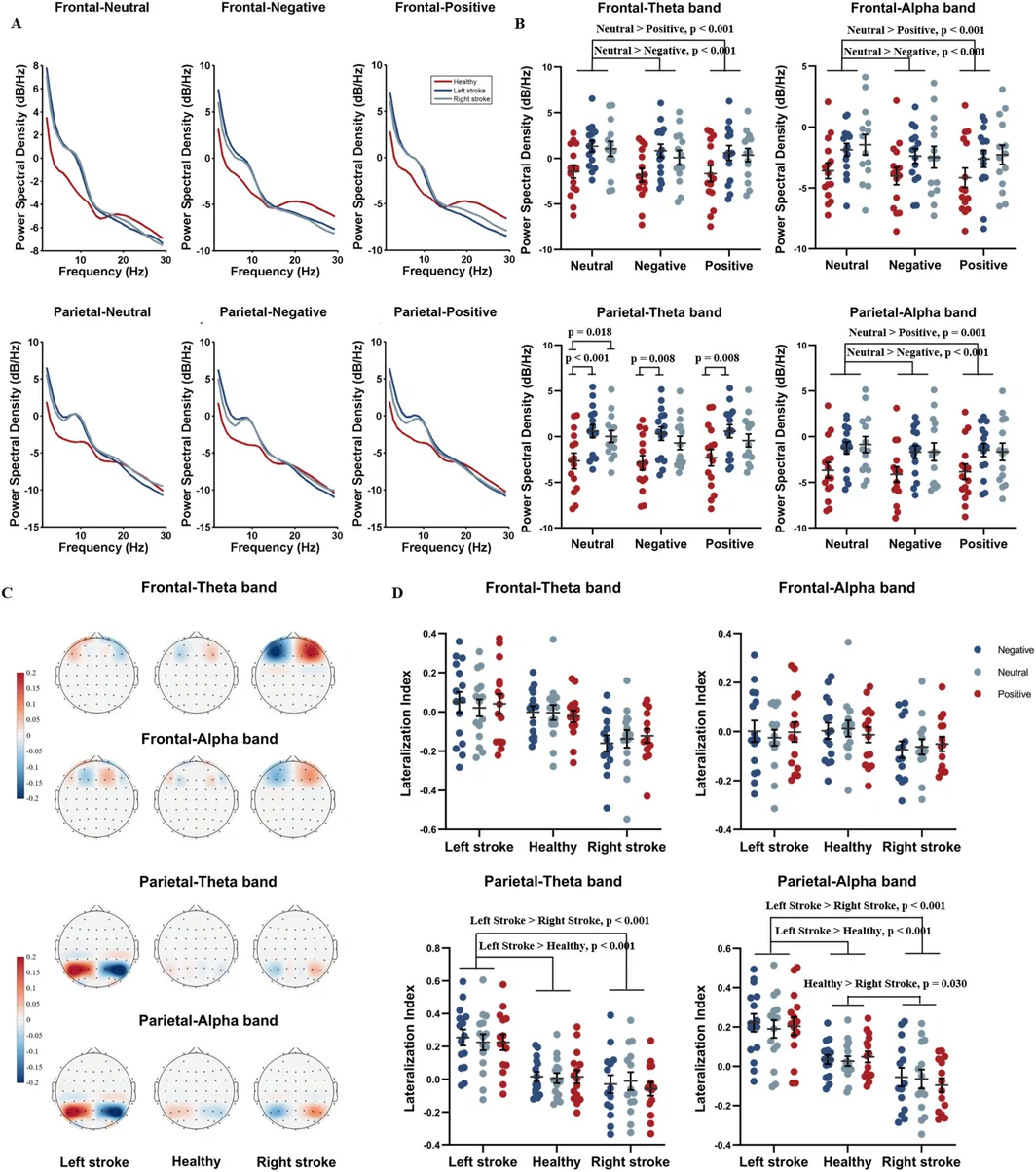
Time‐averaged spectral power and hemispheric lateralization across groups and emotion conditions. (A) Grand average power spectral density plots (1–30 Hz) for the frontal (top row) and parietal (bottom row) regions across neutral, negative, and positive conditions. (B) Scatter plots with means and standard errors for theta (4–7 Hz) and alpha (8–13 Hz) band power in frontal and parietal ROIs. Significance markers indicate post hoc comparisons. (C) Topographic maps showing the distribution of band power lateralization for the Healthy, Left‐stroke, and Right‐stroke groups. The color scale indicates relative power dominance: Red indicates higher power compared to the contralateral site, while blue indicates lower power. Thus, a leftward bias appears as red on the left and blue on the right. (D) Scatter plots of the emotion‐averaged Lateralization Index (LI) for frontal and parietal ROIs in the theta and alpha bands. The central horizontal lines and whiskers represent the mean ± SEM. LI was calculated as (Left − Right) / (Left + Right). Positive values indicate a leftward bias, while negative values indicate a rightward bias.

In the theta and alpha bands, the analyses revealed a robust main effect of emotion, with both positive and negative clips showing desynchronization (i.e., a power decrease) relative to neutral clips in frontal and parietal regions (all p_Holm ≤ 0.001, Figure 2). No significant group main effects were observed for frontal theta, frontal alpha, or parietal alpha power. Furthermore, a significant Group × Emotion interaction was observed for parietal‐theta power (χ^2^(4) = 12.579, p_FDR = 0.027, Table S9). Follow‐up analyses suggested that the L‐stroke group showed higher parietal‐theta power than HC across all emotion conditions (all p_Holm < 0.009), while the R‐stroke group instead exhibited a more emotion‐selective deviation (neutral: p_Holm = 0.018) in parietal‐theta power.

### 3.4 Emotion‐Averaged Lateralization Index

In the frontal region, neither the main effect of emotion, nor the main effect of group, nor the emotion‐by‐group interaction effect reached significance. In contrast, emotion‐averaged parietal LI showed strong group effects in both theta (χ^2^(2) = 17.638, p_FDR < 0.001) and alpha bands (χ^2^(2) = 21.504, p_FDR < 0.001). There were no significant emotion or group × emotion effects in the parietal region (Table S10). Post hoc contrasts revealed that the L‐stroke group showed higher parietal LI than both HC and R‐stroke in theta and alpha bands, whereas the R‐stroke group showed a lower parietal alpha LI than HC (Figure 2). Thus, emotion‐averaged LI primarily reflected hemispheric bias associated with lesion side and was relatively insensitive to emotional modulation.

### 3.5 Inter‐Clip LI Stability

Across the four clips within each emotion category, inter‐clip LI dispersion (SD_trial_) showed an Emotion effect (χ^2^(2) = 9.050, p_FDR = 0.033), whereas detectability (R_det_: χ^2^(2) = 9.512, p_FDR = 0.033) and directional consistency (C_dir_: χ^2^(2) = 9.718, p_FDR = 0.033) were primarily group‐dependent, with no Emotion effects. The Group × Emotion interaction for SDtrial, R_det_, and C_dir_ did not reach significance after FDR correction (Table 2). Thus, inter‐clip LI stability captured stable group differences in the reliability and directional consistency of lateralization across clips, but showed limited evidence for emotion‐specific modulation.

**TABLE 2:** Parietal‐theta inter‐clip lateralization index (LI) stability and within‐clip LI dynamics.

Indicator	Main effect: Group	Main effect: Emotion	Interaction: Group × emotion
**Main analysis at τ = 0.10**			
*Inter‐clip LI stability*			
SD_trial_	Wald χ^2^(2) = 3.599, *p* = 0.165, p_FDR = 0.298	**Wald χ** ^**2**^ **(2) = 9.050, *p* = 0.011, p_FDR = 0.033**	Wald χ^2^(4) = 2.805, *p* = 0.591, p_FDR = 0.665
R_det_	**Wald χ** ^**2**^ **(2) = 9.512, *p* = 0.009, p_FDR = 0.033**	Wald χ^2^(2) = 1.524, *p* = 0.467, p_FDR = 0.600	Wald χ^2^(4) = 10.163, *p* = 0.038, p_FDR = 0.085
C_dir_	**Wald χ** ^**2**^ **(2) = 9.718, *p* = 0.008, p_FDR = 0.033**	Wald χ^2^(2) = 0.555, *p* = 0.758, p_FDR = 0.758	Wald χ^2^(4) = 5.518, *p* = 0.238, p_FDR = 0.357
*Within‐clip LI dynamics*			
R_det_win_	**Wald χ** ^**2**^ **(2) = 15.718, *p* < 0.001, p_FDR** < **0.001**	Wald χ^2^(2) = 6.130, *p* = 0.047, p_FDR = 0.052	**Wald χ** ^**2**^ **(4) = 32.739, *p* < 0.001, p_FDR** < **0.001**
SwitchRate	**Wald χ** ^**2**^ **(2) = 18.850, *p* < 0.001, p_FDR** < **0.001**	**Wald χ** ^**2**^ **(2) = 13.018, *p* = 0.001, p_FDR = 0.002**	**Wald χ** ^**2**^ **(4) = 49.247, *p* < 0.001, p_FDR** < **0.001**
Entropy	**Wald χ** ^**2**^ **(2) = 14.921, *p* < 0.001, p_FDR = 0.001**	Wald χ^2^(2) = 5.126, *p* = 0.077, p_FDR = 0.077	**Wald χ** ^**2**^ **(4) = 14.628, *p* = 0.006, p_FDR = 0.007**
**Sensitivity analysis at τ = 0.05**			
*Inter‐clip LI stability*			
SD_trial_	Wald χ^2^(2) = 3.599, *p* = 0.165, p_FDR = 0.298	Wald χ^2^(2) = 9.050, *p* = 0.011, p_FDR = 0.098	Wald χ^2^(4) = 2.805, *p* = 0.591, p_FDR = 0.665
R_det_	Wald χ^2^(2) = 5.318, *p* = 0.070, p_FDR = 0.167	Wald χ^2^(2) = 2.016, *p* = 0.365, p_FDR = 0.547	Wald χ^2^(4) = 8.527, *p* = 0.074, p_FDR = 0.167
C_dir_	Wald χ^2^(2) = 6.047, *p* = 0.049, p_FDR = 0.167	Wald χ^2^(2) = 0.579, *p* = 0.749, p_FDR = 0.749	Wald χ^2^(4) = 3.579, *p* = 0.466, p_FDR = 0.599
*Within‐clip LI dynamics*			
R_det_win_	**Wald χ** ^**2**^ **(2) = 15.608, *p* < 0.001, p_FDR** < **0.001**	Wald χ^2^(2) = 0.631, *p* = 0.730, p_FDR = 0.730	**Wald χ** ^**2**^ **(4) = 23.406, *p* < 0.001, p_FDR** < **0.001**
SwitchRate	**Wald χ** ^**2**^ **(2) = 17.067, *p* < 0.001, p_FDR** < **0.001**	**Wald χ** ^**2**^ **(2) = 11.921, *p* = 0.003, p_FDR = 0.003**	**Wald χ** ^**2**^ **(4) = 33.459, *p* < 0.001, p_FDR** < **0.001**
Entropy	**Wald χ** ^**2**^ **(2) = 14.475, *p* < 0.001, p_FDR** < **0.001**	Wald χ^2^(2) = 3.283, *p* = 0.194, p_FDR = 0.218	**Wald χ** ^**2**^ **(4) = 16.448, *p* = 0.002, p_FDR = 0.003**
**Sensitivity analysis at τ = 0.15**			
*Inter‐clip LI stability*			
SD_trial_	Wald χ^2^(2) = 3.599, *p* = 0.165, p_FDR = 0.298	**Wald χ** ^**2**^ **(2) = 9.050, *p* = 0.011, p_FDR = 0.024**	Wald χ^2^(4) = 2.805, *p* = 0.591, p_FDR = 0.760
R_det_	**Wald χ** ^**2**^ **(2) = 16.457, *p* < 0.001, p_FDR = 0.001**	Wald χ^2^(2) = 0.211, *p* = 0.900, p_FDR = 0.900	**Wald χ** ^**2**^ **(4) = 13.597, *p* = 0.009, p_FDR = 0.024**
C_dir_	**Wald χ** ^**2**^ **(2) = 20.318, *p* < 0.001, p_FDR** < **0.001**	Wald χ^2^(2) = 0.448, *p* = 0.799, p_FDR = 0.899	Wald χ^2^(4) = 5.936, *p* = 0.204, p_FDR = 0.306
*Within‐clip LI dynamics*			
R_det_win_	**Wald χ** ^**2**^ **(2) = 17.289, *p* < 0.001, p_FDR** < **0.001**	**Wald χ** ^**2**^ **(2) = 6.293, *p* = 0.043, p_FDR = 0.048**	**Wald χ** ^**2**^ **(4) = 49.573, *p* < 0.001, p_FDR** < **0.001**
SwitchRate	**Wald χ** ^**2**^ **(2) = 17.574, *p* < 0.001, p_FDR** < **0.001**	**Wald χ** ^**2**^ **(2) = 6.071, *p* = 0.048, p_FDR = 0.048**	**Wald χ** ^**2**^ **(4) = 65.062, *p* < 0.001, p_FDR** < **0.001**
Entropy	**Wald χ** ^**2**^ **(2) = 15.416, *p* < 0.001, p_FDR** < **0.001**	**Wald χ** ^**2**^ **(2) = 8.057, *p* = 0.018, p_FDR = 0.023**	**Wald χ** ^**2**^ **(4) = 13.529, *p* = 0.009, p_FDR = 0.013**

*Note:* The table summarizes results for Inter‐clip LI stability indicators (SD_trial_, R_det_, C_dir_) and Within‐clip LI dynamic indicators (R_det_win_, SwitchRate, Entropy) at the primary threshold τ = 0.10. Sensitivity analyses at τ = 0.05 and τ = 0.15 are also shown. Bold values indicate statistical significance (p_FDR < 0.05).

### 3.6 Within‐Clip LI Dynamics

Within‐clip LI dynamics showed significant Group × Emotion interactions for within‐clip detectability (R_det_win_) (χ^2^(4) = 32.739, p_FDR < 0.001), switching rate (SwitchRate) (χ^2^(4) = 49.247, p_FDR < 0.001), and entropy (Entropy) (χ^2^(4) = 14.628, p_FDR = 0.007). These results indicate that emotion modulation differed by lesion laterality (Table 2).

**FIGURE 3:**
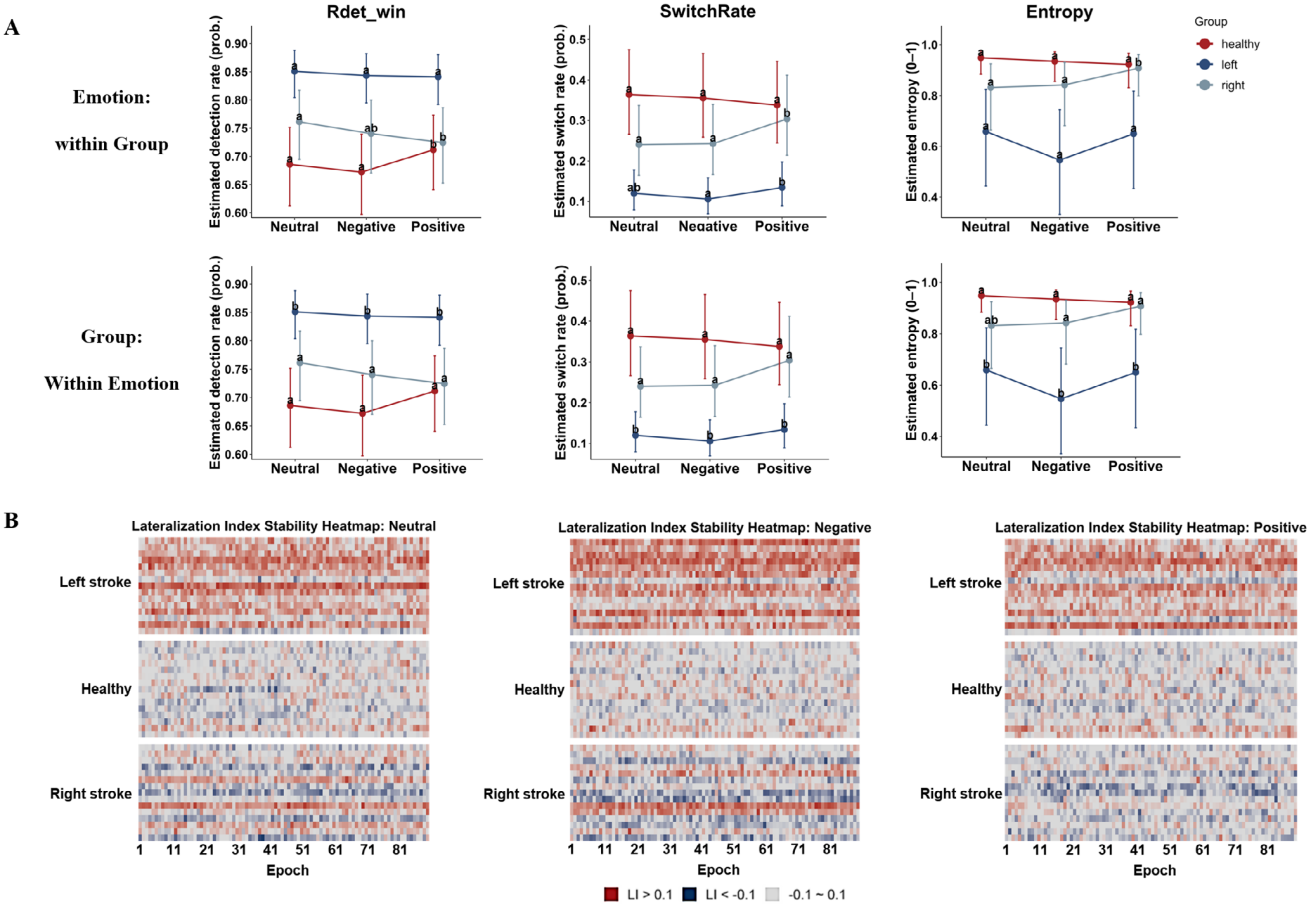
Lesion‐side‐associated differences in within‐clip parietal‐theta lateralization dynamics. (A) Interaction plots for within‐clip lateralization index (LI) dynamic indicators—Detectability (R_det_win_), Switching Rate, and Entropy—show estimated marginal means with 95% confidence intervals. The panels decompose the interaction effects, with the top row illustrating within‐group comparisons across emotion conditions and the bottom row depicting between‐group comparisons within each emotion condition. Significant differences derived from Holm‐adjusted post hoc contrasts are indicated by letters (a, b), where groups or conditions sharing the same letter are not significantly different (*p* > 0.05). (B) Epoch‐resolved LI(t) heatmaps illustrate the temporal evolution of parietal‐theta lateralization across participants for representative clips from each emotion condition. Each row represents one participant, and each column represents one 2‐s epoch. Red indicates left‐lateralized activity (LI > τ), blue indicates right‐dominant epochs (LI < −τ), and gray indicates non‐detectable epochs (|LI| ≤ τ), with τ = 0.10. Darker red or blue shades indicate a greater magnitude of lateralization.

Post hoc analyses showed that, within the HC group, R_det_win_ was higher during positive clips than during neutral and negative clips. SwitchRate and Entropy did not differ across emotions in this group. In the L‐stroke group, SwitchRate was higher during positive clips than during negative clips, with R_det_win_ and Entropy showing no change by emotion. In the R‐stroke group, both SwitchRate and Entropy were higher during positive clips than during neutral and negative clips, while R_det_win_ was lower during positive clips than during neutral clips. Group‐within‐emotion comparisons revealed that, within each emotion condition, the L‐stroke group exhibited higher R_det_win_ and lower SwitchRate than HC and R‐stroke groups. Entropy was lower in the L‐stroke group than in HC across all emotions, and lower than in R‐stroke during positive clips (Figure 3 and Table S11).

Sensitivity analyses generally supported the primary parietal‐theta within‐clip LI dynamic findings after clinical covariate adjustment, brainstem‐case exclusion, retained‐epoch adjustment, and alternative thresholds, although stroke‐only lesion‐category‐adjusted models supported lesion‐side and emotion main effects but not their interaction (Tables S12–S17). Additional LI(t), state occupancy, transition, and secondary ROI × band results are provided in Figures S2–S4 and Table S18.

### 3.7 Arousal and Valence Coupling With Switching Dynamics

#### 3.7.1 Arousal

**FIGURE 4:**
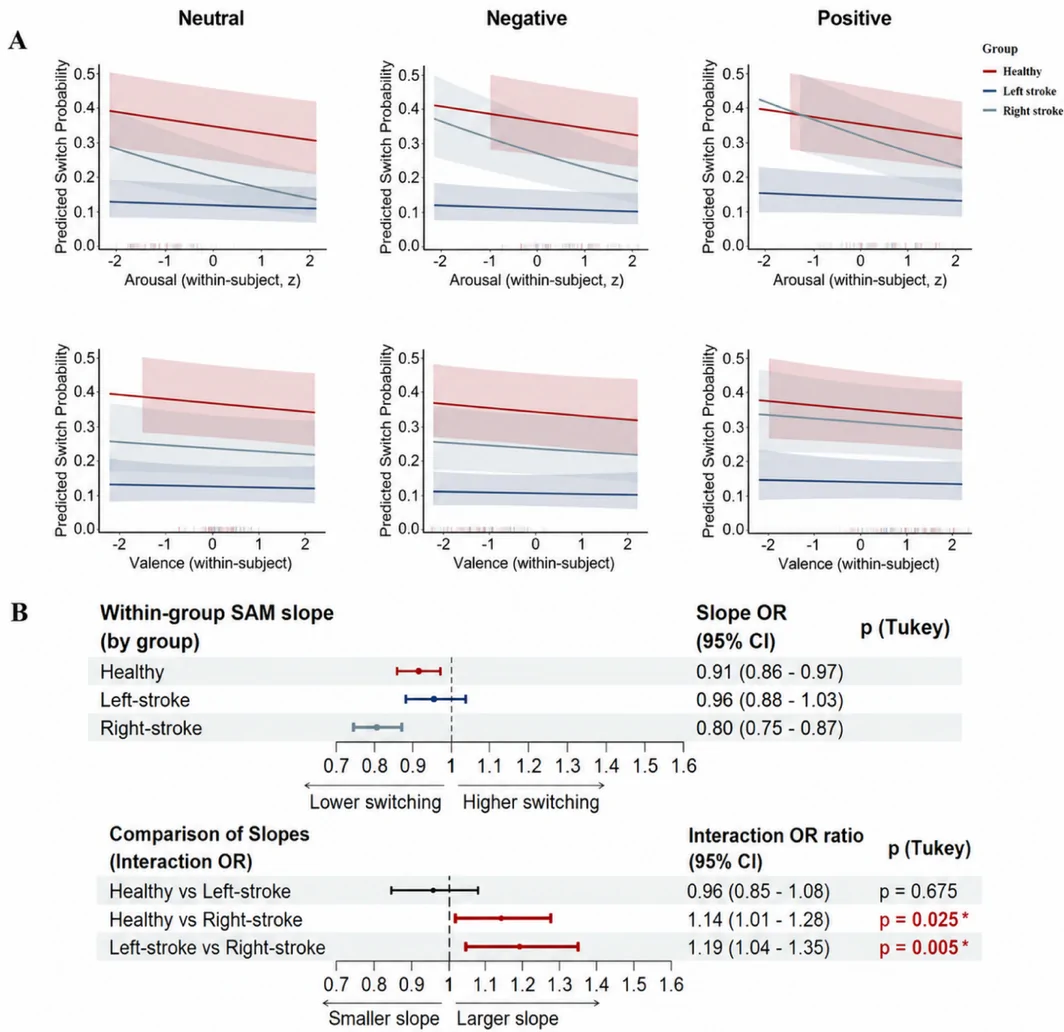
Coupling between subjective emotional experience and neural switching dynamics. (A) Predicted Switching Rate is plotted as a function of within‐subject standardized SAM Arousal (top row) and SAM Valence (bottom row) scores. Solid lines represent fixed‐effect predictions, shaded areas indicate 95% confidence intervals, and rug marks along the x‐axis display the distribution of observed ratings. The plots qualitatively illustrate a steeper negative slope for Arousal in the Right‐stroke group. (B) Forest plots summarize the arousal–switching associations. Higher arousal was associated with lower switching probability, with the strongest effect in R‐stroke. Odds ratios (ORs) are shown for a 1‐SD increase in arousal, where values less than 1 indicate an arousal‐related stabilization effect. Pairwise interaction contrasts showed that this effect was significantly stronger in R‐stroke than in HC and L‐stroke. Error bars represent 95% confidence intervals.

In the primary binomial GLMM, the Group×Arousal interaction was significant (χ^2^(2) = 11.115, *p* = 0.004, Table S19). Higher within‐subject arousal was associated with a lower probability of state switching. This association was most pronounced in R‐stroke (OR = 0.805, 95% CI [0.748, 0.866]) and also present in HC (OR = 0.915, 95% CI [0.860, 0.973]), but not observed in L‐stroke (OR = 0.956, 95% CI [0.883, 1.035]). Gatekept Tukey contrasts indicated a stronger arousal effect in R‐stroke than in HC (*p* = 0.025) and L‐stroke (*p* = 0.005), with no difference between HC and L‐stroke (Figure 4). In summary, R‐stroke showed the largest reduction in switching probability associated with arousal. Further, in a sensitivity analysis, a significant Group × Emotion × Arousal interaction was observed (χ^2^(4) = 12.883, *p* = 0.012).

#### 3.7.2 Valence

In the primary model, there was no evidence that the valence–switching association differed by group (Group × Valence: χ^2^(2) = 0.162, *p* = 0.922, Table S19). In a prespecified sensitivity analysis, an omnibus Group × Emotion × Valence interaction was observed (χ^2^(4) = 29.676, *p* < 0.001).

In exploratory stroke analyses, baseline HAMD and HAMA scores were not significantly associated with parietal‐theta R_det_win_, SwitchRate, or Entropy after FDR correction (all p_FDR > 0.05, Table S16).

## 4 Discussion

In this study, we investigated whether L‐stroke and R‐stroke patients show different patterns of emotional experience and hemispheric lateralization dynamics during naturalistic viewing. Emotional induction was successful across all groups. Stroke patients showed selectively blunted subjective responses to positive clips, especially in the R‐stroke group. Low‐frequency power retained emotion‐related modulation, whereas emotion‐averaged LI mainly reflected lesion‐related hemispheric bias. Within‐clip parietal‐theta dynamics differentiated the stroke groups: The L‐stroke group showed greater state persistence, whereas the R‐stroke group showed greater positive‐context variability and stronger arousal–switching coupling. These patterns were less apparent in time‐averaged measures.

### 4.1 Selective Blunting of Positive Affect After Stroke

Stroke patients frequently undergo emotional changes, such as diminished goal‐directed behavior and lack of interest or pleasure, which may clinically manifest as apathy and anhedonia [34, 35]. Previous studies have reported reduced pleasure and diminished engagement with rewarding experiences after stroke [34, 36]. The study showed that stroke patients exhibited diminished subjective responses to positive film clips, especially in R‐stroke. These findings cannot be fully explained using the classical valence hypothesis; one possible reason for this difference is research methodology. The majority of evidence supporting the classical valence hypothesis mainly relied on responses to brief, static emotional stimuli, whereas the study assessed emotional experience under naturalistic, temporally extended conditions. Under such conditions, positive emotional experience may depend less on initial response generation alone and more on the capacity to sustain engagement and integrate affective meaning over time. Consequently, the more diminished positive emotional responses observed in R‐stroke patients in this study may reflect a greater difficulty in sustaining positive emotional engagement over time than a generalized deficit in generating positive emotions. This interpretation is consistent with evidence implicating the right hemisphere in sustained attentional and affective engagement, but the present data do not permit region‐specific anatomical inferences.

### 4.2 Preserved Low‐Frequency Reactivity but Limited Emotion Sensitivity of Static LI

In all groups, viewing emotional clips (whether positive or negative) resulted in significantly reduced theta and alpha power compared to neutral clips. Given that theta and alpha activity are associated with attentional engagement and cognitive control during emotional processing [37, 38], this suggests that some aspects of stimulus‐related low‐frequency reactivity remain responsive to emotional stimulation after unilateral stroke. However, these power changes were not sufficient to explain the selectively blunted subjective responses to positive clips, particularly in the R‐stroke group. Similarly, emotion‐averaged LI in the parietal region primarily reflected hemispheric bias associated with lesion side, and inter‐clip LI stability indicators provided additional information about the reliability and directional consistency of lateralization across clips, but they did not capture emotion‐specific temporal modulation. Thus, similar average emotional modulation or hemispheric bias may arise from markedly different temporal organizations of lateralized activity, and time‐averaged indicators may therefore obscure transient yet functionally meaningful reorganizations [24, 39], supporting the need for temporally resolved analyses.

### 4.3 Left‐Stroke Group: Patterns Consistent With Greater State Persistence

A prominent dynamic feature after L‐stroke was a rigidity‐like pattern in within‐clip parietal‐theta LI dynamics, which showed increased state detectability, reduced switching, and lower entropy across various emotional conditions. It suggests that lateralized states were not only more frequently detectable but also less likely to switch and less evenly distributed across the available state space over time. Importantly, lower entropy indicates that state occupancy became more concentrated, consistent with a more constrained and less flexible temporal organization of lateralized processing [40]. Given the role that parietal‐theta activity plays in attentional maintenance, cognitive control [41], and the updating of goal‐relevant information under continuous demands [42], this rigidity may reflect diminished capacity to reconfigure attention‐related brain states under continuously changing affective demands. Such a constraint may be particularly consequential for positive emotional experience, which often requires stable attention and continued integration of goal‐relevant contextual information over time [43]. In contrast, negative or threat‐related cues may more readily capture attention through stimulus‐driven mechanisms [44]. On this view, L‐stroke may bias emotional brain dynamics toward excessive persistence, thereby limiting state updating and adaptive reorganization during extended naturalistic viewing.

### 4.4 Right‐Stroke Group: Greater Positive‐Context Variability and Arousal‐Related Switching

In contrast, the R‐stroke group showed a more context‐dependent dynamic pattern. Within‐clip parietal‐theta LI dynamics did not indicate generalized instability across emotions; rather, switching and entropy increased selectively during positive clips. This pattern suggests a context‐specific difficulty in maintaining stable lateralized state organization when positive contexts require sustained integration over time [45]. Brain–behavior coupling further showed that within‐subject increases in arousal were associated with a reduced probability of switching, especially in R‐stroke. Importantly, this stronger coupling does not necessarily indicate better stabilization. Instead, it suggests that the maintenance of stable lateralized states in R‐stroke may depend more heavily on arousal‐related support, especially during positive emotional experience. Positive emotional experience often relies on sustained endogenous engagement and the continued integration of contextual information, whereas negative or threat‐related cues more readily recruit attention through stimulus‐driven processes [43, 44, 46]. If stable state organization in R‐stroke depends more strongly on arousal‐related support, positive contexts may be especially vulnerable, contributing to the blunted positive arousal observed behaviorally. Potential mechanisms may involve disruption of right‐lateralized networks, especially the anterior insula–dorsal anterior cingulate circuitry, which has been proposed to detect salient events and orchestrate control‐relevant engagement by modulating interactions among large‐scale networks [47, 48].

Covariate sensitivity analyses suggested that the dynamic LI findings were not simply explained by baseline anxiety, depressive symptoms, or cognitive performance. However, reduced positive emotional responses may still reflect affective symptoms, cognitive status, or sustained engagement, which should be examined more directly in future studies.

Overall, these findings suggest that hemisphere‐group‐associated differences in the stability–flexibility balance of lateralized brain states may be related to altered emotional processing after stroke. This perspective extends conventional time‐averaged accounts and highlights dynamic lateralization indicators as preliminary candidate electrophysiological features for characterizing post‐stroke emotional dysfunction.

## 5 Limitations

This study has some limitations. Firstly, the small sample size may restrict the ability to detect minor effects and complex interactions, with lesion anatomy varying within hemisphere groups despite broad lesion‐location stratification and sensitivity analyses. Secondly, the study used a common average reference for EEG analyses without testing alternative references. Thirdly, while LI normalization reduces dependence on absolute power, thresholded LI dynamics might still be influenced by signal‐to‐noise variability. The cross‐sectional design limits causal inference and the tracking of temporal changes, necessitating longitudinal studies to determine whether these characteristics are temporary or lasting, and whether they relate to recovery or treatment response. Lastly, although participants viewed the same validated film clips in a pseudo‐randomized order, variations in clip features could affect LI dynamics. Future research should use more balanced stimuli, eye tracking, and additional physiological monitoring.

## 6 Conclusions

Our findings suggest that the L‐stroke and R‐stroke groups differed in the temporal dynamics of lateralized brain states during naturalistic viewing. Time‐averaged spectral and LI measures mainly captured low‐frequency reactivity and lesion‐related hemispheric bias, whereas within‐clip parietal‐theta LI dynamics showed greater state persistence in the L‐stroke group and greater positive‐context variability in the R‐stroke group. These preliminary patterns require replication in larger, anatomically characterized longitudinal cohorts.

## Author Contributions

J.L. and M.H. conceived the study. J.L., Z.C., X.L., and M.H. contributed to the study design. M.H., Y.T., Y.L., and X.C. collected the clinical data. M.H. processed the data and conducted the main analysis. M.H. wrote the original manuscript draft. J.L. critically revised the manuscript and contributed the most important intellectual content. All authors revised the manuscript for important intellectual content and approved the final version.

## Funding

This work was supported by the National Key R&D Program of China (2020YFC2005700) and the National Natural Science Foundation of China (62276115).

## Disclosure

During the preparation of this work, the authors used ChatGPT to improve the language and readability of the manuscript. After using this tool, the authors reviewed and edited the content as needed and take full responsibility for the content of the published article.

## Conflicts of Interest

The authors declare no conflicts of interest.

## Supporting information


**Figure S1:** Schematic illustration of the experimental design.
**Figure S2:** Representative epoch‐resolved parietal‐theta LI(t) traces across emotion conditions.
**Figure S3:** State occupancy proportions of parietal‐theta lateralization states.
**Figure S4:** Parietal‐theta tri‐state transition probability matrices during positive clips.
**Table S1:** Film clip information and selected descriptive audiovisual features.
**Table S2:** Descriptive statistics and permutation ANOVA results for selected audiovisual features by emotion category.
**Table S3:** EEG epoch‐retention summary after artifact rejection by group and emotion.
**Table S4:** Patient‐level lesion characteristics and broad lesion‐category classification.
**Table S5:** Distribution of broad lesion categories by hemisphere.
**Table S6:** Descriptive statistics and mixed‐effects model results for Self‐Assessment Manikin (SAM) ratings.
**Table S7:** Sensitivity analysis of SAM ratings adjusting for baseline MoCA, HAMA, and HAMD scores.
**Table S8:** Covariate‐adjusted simple contrasts for positive SAM ratings.
**Table S9:** Descriptive statistics and statistical results for Power Spectral Density (PSD).
**Table S10:** Descriptive statistics and statistical results for emotion‐averaged Lateralization Index (LI).
**Table S11:** Model‐based effect estimates and 95% confidence intervals for primary within‐clip parietal‐theta LI dynamic outcomes at τ = 0.10.
**Table S12:** Sensitivity analysis of primary parietal‐theta within‐clip LI dynamics adjusting for baseline MoCA, HAMA, and HAMD scores.
**Table S13:** Brainstem‐exclusion sensitivity analysis for behavioral ratings and primary parietal‐theta within‐clip LI dynamics.
**Table S14:** Exploratory stroke‐only sensitivity analysis of primary parietal‐theta within‐clip LI dynamics after excluding brainstem lesions and adjusting for lesion category.
**Table S15:** Sensitivity analysis of parietal‐theta within‐clip LI dynamics using data‐driven thresholds.
**Table S16:** Exploratory stroke‐only correlations between HAMD/HAMA scores and primary parietal‐theta dynamic LI metrics.
**Table S17:** Robustness analyses of primary parietal‐theta dynamic LI outcomes adjusting for retained epoch number.
**Table S18:** Secondary analyses of inter‐clip LI stability and within‐clip LI dynamics in other ROI × band combinations.
**Table S19:** Type III Wald χ2 tests for brain–behavior coupling models of SwitchRate.

## Data Availability

The data associated with this study will be made available from the corresponding author upon reasonable request. Because the dataset contains clinical and electrophysiological data from human participants, public deposition is subject to ethical and privacy restrictions. The analysis code used in this study will be made available from the corresponding author upon reasonable request.
