# Supplementary material for: Dynamic Hemispheric Lateralization of Naturalistic Emotional Processing After Unilateral Stroke: An EEG Study
Source: CNS Neurosci Ther. 2026 Aug 20;32(8):e71096. doi: 10.1002/cns.71096 (PMC13492182; doi:10.1002/cns.71096)
Supplement: Supplementary file 1 [file CNS-32-e71096-s001.docx]

**Supplementary Material**

**Supplementary Methods**

**Experimental Procedure**

The emotional film clips were adapted from the SEED paradigm ^1,2^. Participants were seated in a comfortable chair within a quiet, electromagnetically shielded room and instructed to minimize movement while watching the clips naturally. The experimental procedure for each trial was as follows (Figure S1): (1) a 3-s on-screen cue (“Please prepare to watch”); (2) presentation of a film clip (approximately 3–4 min each); (3) a 45-s self-assessment period; and (4) a 1-min rest period before the next trial. During self-assessment, participants rated their subjective experience using the 9-point Self-Assessment Manikin (SAM) scale for valence (1 = very unpleasant, 9 = very pleasant) and arousal (1 = very calm, 9 = very excited).

**EEG acquisition**

EEG was recorded using a 64-channel Ag/AgCl cap arranged according to the 10–20 system and amplified with a Neuroscan Synamps2 amplifier (Neurosoft, Inc., Sterling, VA, United States). Recordings were collected at a sampling rate of 1000 Hz, with electrode impedance kept below 5 kΩ and online bandpass filtering from 0.01 to 100 Hz. A ground electrode was placed between the FPZ and FZ electrodes, and reference electrodes were positioned on the bilateral mastoids.

**Assessment of Inter-Clip Lateralization Stability**

To quantify the stability of hemispheric lateralization across the four clips within each emotion category, we derived three complementary LI-based indicators for each Participant × Emotion × ROI × Frequency Band from the four clip-level lateralization index (LI) values, capturing dispersion in magnitude, reliability of lateralization emergence, and directional consistency.

**(i) Inter-clip dispersion (SD_trial_):** Variability in lateralization magnitude was quantified as the standard deviation of clip-level LI values across the four clips (LI_1_, LI_2_, LI_3_, LI_4_). Due to the typical skewness of dispersion measures, SD_trial_ was log-transformed, *log(SD+ϵ)*, where ϵ is a small constant to avoid undefined values.

${SD}_{trial}=log( SD({LI}_{1}, {LI}_{2}, {LI}_{3}, {LI}_{4})+\varepsilon$*)*

**(ii) Inter-clip Detectability (R_det_):** Detectability was defined as the proportion of clips in which lateralization magnitude exceeded the band threshold (|LI|>τ). This metric reflects the reliability with which a lateralized state emerged at the clip level.

$$R_{det}=\frac{k_{det}}{4}, k_{det}=\sum_{c=1}^{4} Ⅱ(|{LI}_{c}|>\tau)$$

*Ⅱ* denotes an indicator function, which takes the value 1 if the condition inside the parentheses is satisfied and 0 otherwise. *LI_c_* denotes clip-level LI for clip c.

**(iii) Directional consistency (C_dir_):** To quantify the stability of lateralization direction independent of magnitude, clip-level LI values were mapped to a tri-state sign variable {−1/0/+1} using the band rule (0 for |LI|≤τ, and ±1 for |LI|>τ according to sign). C_dir_ was computed as the proportion of detectable clips (non-zero states) that matched the modal non-zero sign across the four clips.

$$n_{+}=\sumⅡ({LI}_{c}>\tau),n_{-}=\sumⅡ({LI}_{C}<-\tau)$$

$$C_{dir}=\frac{max(n_{+},n_{-})}{n_{+}+n_{-}}$$

The primary analyses used τ = 0.10 ^3^, with sensitivity analyses at τ = 0.05 and τ = 0.15.

**Quantification of Within-Clip LI(t) Dynamics**

To capture the temporal evolution of hemispheric lateralization within each clip, epoch-resolved LI(t) series were discretized into a tri-state sequence {−1/0/+1} using the same band rule described above^4,5^. For each Participant × Emotion × Clip × ROI × Frequency Band, three within-clip LI dynamic indicators were computed.

**(i) Within-clip detectability (R_det_win_)**. Within-clip detectability was defined as the proportion of epochs exhibiting a detectable lateralized state (|LI|>τ), modeled as binomial counts (*k_det_* out of *n* epochs). *LI_(t)_* denotes epoch-level LI.

$$R_{\text{det\_win}}=\frac{k_{\text{det}}}{n},k_{\text{det}}=\sum_{t=1}^{n} Ⅱ(|LI(t)|>\tau)$$

**(ii) Switching rate (SwitchRate):** To index state transitions independent of transient non-detectable periods, switching was quantified on the subsequence of detectable epochs only (zeros excluded), thereby reducing spurious “switches” caused by threshold crossings near τ^4,5^. A switch was defined as a sign reversal between consecutive detectable epochs, modeled as the number of switches (*k_switch_*) out of the number of valid transitions (*k_comp_*).

$$k_{\text{switch}}=\sum_{i=2}^{m} Ⅱ\left( \tilde{d}_{i}\neq\tilde{d}_{i-1} \right)$$

$$k_{\text{comp}}=k_{\text{det}}-1$$

$$SwitchRate=\frac{k_{switch}}{k_{comp}}$$

Switching was counted as sign reversals between consecutive elements in $\tilde{d}_{i}$.

**(iii) State complexity (Entropy):** State complexity was quantified as the normalized Shannon entropy of the tri-state occupancy distribution, yielding values from 0 (occupancy concentrated in a single state) to 1 (uniform occupancy across all three states)^5^.

$$Entropy=-\frac{\sum_{s} p_{s}{log}_{2} (p_{s})}{{log}_{2} 3}$$

*P_s_* is the occupancy probability of state *s*.

Consistent with inter-clip stability, the primary threshold was set at τ = 0.10^3^ with sensitivity analyses at τ = 0.05 and τ = 0.15. Additionally, data-driven sensitivity analyses were performed using empirical |LI| thresholds derived from hemisphere-swapping permutation tests in the healthy-neutral condition. The thresholds corresponding to the 90th, 95th, and 99th percentiles of the null |LI| distribution were τ_90 = 0.273, τ_95 = 0.342, and τ_99 = 0.497, respectively. These thresholds were used to evaluate the robustness of the findings across increasingly stringent null-based cutoffs.

**Statistical Analysis**

Statistical analyses were performed using R (version 4.4.2). Continuous variables are reported as mean (standard deviation) or median (interquartile range) and categorical variables as numbers (percentage). Group differences at baseline were assessed using the Kruskal-Wallis H tests for continuous variables, and chi-square tests or Fisher's exact test for categorical variables. In addition, between-stroke-group comparisons (L-stroke vs R-stroke) were performed for type of stroke using Fisher’s exact test and for time post-stroke using the Mann-Whitney U test.

The primary EEG analysis focused on within-clip parietal-theta LI dynamics at τ = 0.10 ^3^. PSD, emotion-averaged LI, and inter-clip LI stability were treated as secondary analyses, whereas other ROI × band combinations, alternative thresholds, lesion-category analyses, and clinical-scale correlations were considered exploratory or sensitivity analyses.

SAM ratings, PSD, and emotion-averaged LI were analyzed using mixed-effects models that incorporated fixed effects for Group, Emotion, and their interaction, with participant modeled as a random intercept. Inter-clip LI stability indicators were analyzed at the Participant × Emotion level with participant as a random intercept. For within-clip LI dynamic indicators, Clip was additionally included as a crossed random intercept. Continuous outcomes were analyzed using linear mixed-effects models. We modeled dispersion on the log scale and adjusted entropy with boundary correction before logit transformation. Detectability and switching outcomes were analyzed using binomial generalized linear mixed-effects models (GLMMs), with events per trial as the response variable.

Fixed effects in the mixed-effects models were evaluated using Type III Wald chi-square tests. Multiplicity was controlled using Benjamini-Hochberg false discovery rate (FDR) within prespecified families (ROI×band features and/or indicators within τ and effect), followed by Holm-adjusted post-hoc contrasts when needed. Brain-behavior coupling analyses focused a priori on parietal-theta switching rate: within-subject standardized SAM scores were modeled via a Group×SAM interaction (controlling for Emotion and Group × Emotion), with Tukey-adjusted pairwise slope comparisons performed if the interaction was significant. Additional clinical, lesion-related, epoch-retention, and threshold-sensitivity analyses are described in the Supplementary Methods. All tests were two-sided with α = 0.05.

**Sensitivity Analyses**

To evaluate potential confounding by baseline cognitive performance and affective symptoms, the key behavioral models and primary parietal-theta within-clip LI dynamic models were re-estimated with baseline MoCA, HAMA, and HAMD scores as covariates. For SAM ratings, covariate-adjusted simple contrasts were additionally estimated during positive clips to directly examine group differences in reduced positive valence and arousal. Holm correction was applied across the three group contrasts within each SAM outcome.

Based on clinical neuroimaging descriptions, lesions were categorized as cortical, subcortical, or brainstem. Lesion-related sensitivity analyses were performed by excluding brainstem cases. In additional stroke-only models, brainstem cases were excluded and broad lesion category (cortical versus subcortical) was entered as a fixed covariate. Because of the reduced stroke-only sample size and the broad nature of this lesion classification, these lesion-category-adjusted models were considered exploratory. Exploratory stroke-only Spearman correlations were used to test associations between baseline HAMD/HAMA scores and participant-level parietal-theta dynamic LI measures for positive clips and emotion-averaged measures, with FDR correction applied across the affective-symptom correlation family. To visualize the dynamic LI state structure, state-occupancy proportions and row-normalized transition probability matrices were summarized from the tri-state LI sequences. Robustness analyses further adjusted the primary parietal-theta dynamic LI models for retained epoch number. Threshold sensitivity was assessed using alternative fixed thresholds and data-driven thresholds derived from permutation testing.

**Supplementary References**

1.	Zheng WL, Lu BL. Investigating Critical Frequency Bands and Channels for EEG-Based Emotion Recognition with Deep Neural Networks. *IEEE Transactions on Autonomous Mental Development*. 2015;7(3):162-175. doi:10.1109/TAMD.2015.2431497

2.	Duan RN, Zhu JY, Lu BL. Differential entropy feature for EEG-based emotion classification. 2013:81-84.

3.	Seghier ML. Laterality index in functional MRI: methodological issues. *Magn Reson Imaging*. Jun 2008;26(5):594-601. doi:10.1016/j.mri.2007.10.010

4.	von Wegner F, Tagliazucchi E, Laufs H. Information-theoretical analysis of resting state EEG microstate sequences - non-Markovianity, non-stationarity and periodicities. *Neuroimage*. Sep 2017;158:99-111. doi:10.1016/j.neuroimage.2017.06.062

5.	von Wegner F, Knaut P, Laufs H. EEG Microstate Sequences From Different Clustering Algorithms Are Information-Theoretically Invariant. *Front Comput Neurosci*. 2018;12:70. doi:10.3389/fncom.2018.00070

**Fig S1. Schematic illustration of the experimental design**

The sequence of a single trial included a hint (3 s), film clip presentation (3-4 min), self-assessment (SAM, 45 s), and a rest period (1 min).

**Fig S2.** **Representative epoch-resolved parietal-theta LI(t) traces across emotion conditions**

**Note:** Epoch-resolved parietal-theta LI(t) traces are shown for illustrative participants from the HC, L-stroke, and R-stroke groups across neutral, negative, and positive conditions. The grey shaded area indicates the non-detectable zone defined by the primary threshold, |LI| ≤ 0.10. These examples are provided to visualize temporal LI fluctuations and inter-individual variability; formal statistical inference was based on the full mixed-effects models rather than on these illustrative traces. HC = healthy control; L-stroke = left-hemisphere stroke; R-stroke = right-hemisphere stroke; LI = lateralization index.

**Fig S3.** **State occupancy proportions of parietal-theta lateralization states**

**Note:** Stacked bars show the proportion of epochs classified as left-lateralized (+1, LI > 0.10), non-detectable (0, |LI| ≤ 0.10), or right-lateralized (−1, LI < −0.10) for each Group × Emotion condition. State occupancy was first calculated at the Participant × Emotion × Clip level and then averaged within participants before group-level visualization. HC = healthy control; L-stroke = left-hemisphere stroke, R-stroke = right-hemisphere stroke, LI = lateralization index.

**Fig S4.** **Parietal-theta tri-state transition probability matrices during positive clips**

**Note:** Row-normalized 3 × 3 matrices show the probability of moving from one LI state at epoch t to another state at epoch t + 1 during positive clips. Rows indicate the state at epoch t, and columns indicate the state at epoch t + 1. Row and column states are ordered identically; therefore, diagonal values indicate state persistence, whereas off-diagonal values indicate transitions between states. Matrices are shown for positive clips because the strongest group-dependent dynamic LI effects were observed under this condition. These tri-state matrices provide a descriptive visualization of consecutive epoch-to-epoch transitions and are complementary to, rather than mathematically identical to, SwitchRate, which was calculated after excluding non-detectable epochs. HC = healthy control; L-stroke = left-hemisphere stroke; R-stroke = right-hemisphere stroke; LI = lateralization index.

**Table S1.** **Film clip information and selected descriptive audiovisual features**

| **Clip No.** | **Source film/documentary** | **Emotion category** | **Start time** | **End time** | **Duration, s** | **Mean luminance** | **Audio RMS amplitude** |
| --- | --- | --- | --- | --- | --- | --- | --- |
| 1 | Lost in Thailand | positive | 00:06:13 | 00:10:11 | 238 | 71.21 | 0.01 |
| 2 | Aftershock | negative | 00:20:10 | 00:23:35 | 205 | 34.19 | 0.02 |
| 3 | Back to 1942 | negative | 00:49:58 | 00:54:00 | 242 | 46.88 | 0.02 |
| 4 | World Heritage in China EP2 | neutral | 00:10:40 | 00:13:44 | 184 | 83.39 | 0.02 |
| 5 | Lost in Thailand | positive | 01:05:10 | 01:08:29 | 199 | 80.68 | 0.03 |
| 6 | Back to 1942 | negative | 02:01:21 | 02:05:21 | 240 | 73.76 | 0.02 |
| 7 | World Heritage in China EP13 | neutral | 00:02:55 | 00:06:35 | 220 | 81.05 | 0.02 |
| 8 | Just Another Pandora's Box | positive | 00:11:32 | 00:15:33 | 241 | 65.71 | 0.01 |
| 9 | World Heritage in China EP13 | neutral | 00:10:41 | 00:14:38 | 237 | 84.38 | 0.02 |
| 10 | World Heritage in China EP30 | neutral | 00:05:36 | 00:09:36 | 240 | 79.69 | 0.01 |
| 11 | Just Another Pandora's Box | positive | 00:35:00 | 00:39:02 | 242 | 98.57 | 0.02 |
| 12 | Aftershock | negative | 01:48:53 | 01:52:18 | 205 | 46.92 | 0.02 |

**Note:** Mean luminance was calculated from frame-wise grayscale intensity. Audio RMS amplitude was calculated from the extracted audio waveform and is provided as a descriptive index of overall auditory intensity. These features were summarized to improve stimulus reproducibility rather than to indicate complete matching across emotion categories. RMS = root mean square.

**Table S2. Descriptive statistics and permutation ANOVA results for selected audiovisual features by emotion category**

| **Audiovisual features** | **Emotion category** | **Mean ± SD** | **Median** | **Omnibus permutation ANOVA** |
| --- | --- | --- | --- | --- |
|  | neutral | 220.25 ± 25.72 | 228.50 |  |
| **Duration, s** | positive | 230.00 ± 20.74 | 239.50 | F(2, 9) = 0.199, p_perm = 0.862, η² = 0.042 |
|  | negative | 223.00 ± 20.80 | 222.50 |  |
|  | neutral | 82.13 ± 2.14 | 82.22 |  |
| **Mean luminance** | positive | 79.04 ± 14.41 | 75.95 | **F(2, 9) = 7.479, p_perm = 0.017, η² = 0.624** |
|  | negative | 50.44 ± 16.66 | 46.90 |  |
|  | neutral | 0.02 ± 0.01 | 0.02 |  |
| **Audio RMS amplitude** | positive | 0.02 ± 0.01 | 0.02 | F(2, 9) = 0.214, p_perm = 1.000, η² = 0.045 |
|  | negative | 0.02 ± 0.01 | 0.02 |  |

**Note:** Values are presented as mean ± standard deviation and median. For each selected audiovisual feature, a one-way permutation ANOVA was conducted with emotion category as the between-clip factor while preserving the original group sizes of four clips per emotion category. The reported p values are uncorrected exploratory permutation p values, and effect size is reported as η². Mean luminance was calculated from frame-wise grayscale intensity. Audio RMS amplitude was calculated from the extracted audio waveform and is provided as a descriptive index of overall auditory intensity rather than auditory variability. Because the omnibus test for mean luminance was significant, post hoc pairwise permutation tests were conducted for this feature. Negative clips showed lower mean luminance than neutral clips, mean difference = −31.69, p_perm = 0.029. The difference between negative and positive clips did not reach statistical significance, mean difference = −28.60, p_perm = 0.086, and no significant difference was observed between neutral and positive clips, mean difference = 3.09, p_perm = 0.743. These analyses were performed to improve stimulus transparency and reproducibility. RMS = root mean square. Bold values indicate statistical significance (p_perm < 0.05).

**Table S3. EEG epoch-retention summary after artifact rejection by group and emotion**

| **Group** | **Emotion** | **Participants, n** | **Retained epochs, mean ± SD** | **Range** |
| --- | --- | --- | --- | --- |
| Healthy | Negative | 15 | 392.1 ± 41.6 | 306–442 |
| Healthy | Neutral | 15 | 370.3 ± 54.9 | 282–437 |
| Healthy | Positive | 15 | 394.2 ± 37.2 | 334–451 |
| Left Stroke | Negative | 15 | 373.1 ± 41.1 | 286–435 |
| Left Stroke | Neutral | 15 | 374.8 ± 42.1 | 307–438 |
| Left Stroke | Positive | 15 | 411.3 ± 38.1 | 323–458 |
| Right Stroke | Negative | 15 | 337.8 ± 57.5 | 270–431 |
| Right Stroke | Neutral | 15 | 344.5 ± 41.6 | 273–410 |
| Right Stroke | Positive | 15 | 358.9 ± 58.1 | 279–451 |

**Note:** Retained epochs were calculated after automatic artifact rejection using an amplitude threshold of ±100 μV. For each participant and emotion condition, retained epochs were summed across the four clips belonging to that emotion category. Each epoch was 2 s in duration.

**Table S4.** **Patient-level lesion characteristics and broad lesion-category classification**

| **ID** | **Stroke Type** | **Hemisphere** | **Lesion Location** | **Lesion Size** | **Secondary Lesion Classification** |
| --- | --- | --- | --- | --- | --- |
| 01 | Hemorrhagic | Right | Thalamus | 9 mL | Subcortical |
| 02 | Ischemic | Right | Temporo-parietal lobe | N/A | Cortical |
| 03 | Ischemic | Right | Frontal-temporal lobe | N/A | Cortical |
| 04 | Ischemic | Left | Basal ganglia | N/A | Subcortical |
| 05 | Hemorrhagic | Left | Basal ganglia | 15 mL | Subcortical |
| 06 | Ischemic | Left | Frontoparietal lobe | N/A | Cortical |
| 07 | Ischemic | Right | Basal ganglia | N/A | Subcortical |
| 08 | Hemorrhagic | Right | Basal ganglia | 17 mL | Subcortical |
| 09 | Ischemic | Right | Basal ganglia | N/A | Subcortical |
| 10 | Ischemic | Left | Frontoparietal lobe | N/A | Cortical |
| 11 | Hemorrhagic | Right | Pons | 0.8 mL | Brainstem |
| 12 | Ischemic | Right | Basal ganglia | N/A | Subcortical |
| 13 | Ischemic | Right | Basal ganglia | N/A | Subcortical |
| 14 | Ischemic | Right | Frontal-temporal lobe | N/A | Cortical |
| 15 | Ischemic | Left | Frontal-temporal lobe | N/A | Cortical |
| 16 | Ischemic | Left | Basal ganglia | N/A | Subcortical |
| 17 | Ischemic | Left | Basal ganglia | N/A | Subcortical |
| 18 | Ischemic | Right | Basal ganglia | N/A | Subcortical |
| 19 | Hemorrhagic | Left | Pons | 1.5 mL | Brainstem |
| 20 | Hemorrhagic | Left | Basal ganglia | 26 mL | Subcortical |
| 21 | Ischemic | Right | Frontoparietal lobe | N/A | Cortical |
| 22 | Hemorrhagic | Left | Thalamus | 10 mL | Subcortical |
| 23 | Ischemic | Left | Basal ganglia | N/A | Subcortical |
| 24 | Hemorrhagic | Right | Basal ganglia | 30.5 mL | Subcortical |
| 25 | Ischemic | Left | Basal ganglia | N/A | Subcortical |
| 26 | Ischemic | Left | Frontal-temporal lobe | N/A | Cortical |
| 27 | Ischemic | Left | Basal ganglia | N/A | Subcortical |
| 28 | Ischemic | Right | Basal ganglia | N/A | Subcortical |
| 29 | Hemorrhagic | Right | Basal ganglia | 6 mL | Subcortical |
| 30 | Hemorrhagic | Left | Basal ganglia | 12 mL | Subcortical |

**Note:** Lesion locations and lesion-size information were extracted from clinical neuroimaging reports. Lesions were secondarily classified into cortical, subcortical, or brainstem categories for descriptive and sensitivity analyses. Lesion size is reported when available; N/A indicates that lesion-size information was not available in the clinical report, mainly for ischemic lesions. mL = milliliter; N/A = not available.

**Table S5.** **Distribution of broad lesion categories by hemisphere**

| **Secondary lesion category** | **Total N** | **Right hemisphere** | **Left hemisphere** |
| --- | --- | --- | --- |
| Cortical | 8 | 4 | 4 |
| Subcortical | 20 | 10 | 10 |
| Brainstem | 2 | 1 | 1 |
| Total | 30 | 15 | 15 |

**Note:** Lesion categories were derived from clinical neuroimaging reports. Cortical lesions included frontotemporal, frontoparietal, and temporoparietal lesions; subcortical lesions included basal ganglia and thalamic lesions; brainstem lesions included pontine lesions. Broad lesion-category distribution was balanced between the left- and right-hemisphere stroke groups.

**Table S6. Descriptive statistics and mixed-effects model results for Self-Assessment Manikin (SAM) ratings**

| **Emotion** | **Healthy** | **Left Stroke** | **Right Stroke** | **Main Effect: Group** | **Main Effect: Emotion** | **Interaction Group×Emotion** |
| --- | --- | --- | --- | --- | --- | --- |
| **Valence** | | | | | | |
| Negative | 2.80 ± 1.09 | 2.77 ± 0.90 | 3.04 ± 0.53 |  |  |  |
| Neutral | 5.35 ± 0.40 | 5.39 ± 0.27 | 5.17 ± 0.29 | Wald χ²(2) = 4.176, p = 0.124, p_FDR = 0.124 | **Wald χ²(2) = 208.460, p < 0.001, p_FDR < 0.001** | **Wald χ²(4) = 13.150, p = 0.011, p_FDR = 0.013** |
| Positive | 7.33 ± 1.02 | 6.59 ± 0.81 | 6.19 ± 0.73 |  |  |  |
| **Arousal** | | | | | | |
| Negative | 5.73 ± 1.24 | 5.38 ± 1.40 | 4.98 ± 1.21 |  |  |  |
| Neutral | 2.10 ± 0.77 | 1.56 ± 0.64 | 1.39 ± 0.44 | **Wald χ²(2) = 11.180, p = 0.004, p_FDR = 0.006** | **Wald χ²(2) = 174.845, p < 0.001, p_FDR < 0.001** | **Wald χ²(4) = 16.723, p = 0.002, p_FDR = 0.004** |
| Positive | 6.18 ± 1.57 | 5.05 ± 1.38 | 3.61 ± 1.32 |  |  |  |

**Note:** Data are presented as mean ± standard deviation. Main effects and interaction results are derived from mixed-effects models. Bold values indicate statistical significance (p_FDR < 0.05).

**Table S7. Sensitivity analysis of SAM ratings adjusting for baseline MoCA, HAMA, and HAMD scores**

| **SAM** | **Main Effect: Group** | **Main Effect: Emotion** | **Interaction: Group × Emotion** | **Covariates (MoCA, HAMA, HAMD)** |
| --- | --- | --- | --- | --- |
| Valence | Wald χ²(2) = 2.004, p = 0.367, p_FDR = 0.367 | **Wald χ²(2) = 466.911, p < 0.001, p_FDR < 0.001** | **Wald χ²(4) = 18.922, p < 0.001, p_FDR = 0.002** | Wald χ²(3) = 3.204, p = 0.361, p_FDR = 0.367 |
| Arousal | **Wald χ²(2) = 23.265, p < 0.001, p_FDR < 0.001** | **Wald χ²(2) = 302.535, p < 0.001, p_FDR < 0.001** | **Wald χ²(4) = 17.311, p = 0.002, p_FDR = 0.003** | Wald χ²(3) = 3.579, p = 0.311, p_FDR = 0.367 |

**Note:** Models included fixed effects for Group, Emotion, Group × Emotion, and baseline MoCA, HAMA, and HAMD scores. The covariate column represents the joint 3-df test of the three baseline covariates. p_FDR values were Benjamini-Hochberg corrected within this covariate-adjusted behavioral sensitivity-analysis family. Bold values indicate statistical significance (p_FDR < 0.05). SAM = Self-Assessment Manikin; MoCA = Montreal Cognitive Assessment; HAMD = Hamilton Depression Rating Scale; HAMA = Hamilton Anxiety Rating Scale.

**Table S8. Covariate-adjusted simple contrasts for positive SAM ratings**

| **SAM** | **Contrast** | **Estimate** | **95% CI** | **p** | **p_Holm** |
| --- | --- | --- | --- | --- | --- |
| Valence | HC vs L-stroke | 0.705 | [0.016, 1.395] | **0.014** | **0.029** |
| Valence | HC vs R-stroke | 1.054 | [0.342, 1.766] | **< 0.001** | **0.001** |
| Valence | L-stroke vs R-stroke | 0.348 | [-0.313, 1.010] | 0.204 | 0.204 |
| Arousal | HC vs L-stroke | 1.261 | [0.156, 2.367] | **0.006** | **0.008** |
| Arousal | HC vs R-stroke | 2.520 | [1.367, 3.674] | **< 0.001** | **< 0.001** |
| Arousal | L-stroke vs R-stroke | 1.259 | [0.216, 2.302] | **0.004** | **0.008** |

**Note:** Simple contrasts were derived from covariate-adjusted mixed-effects models including Group, Emotion, Group × Emotion, baseline MoCA, HAMA, and HAMD scores, with participant as a random intercept. Only contrasts during positive clips are shown. Estimates represent adjusted between-group differences in SAM ratings, with positive values indicating higher ratings in the first group of each contrast. p_Holm values were Holm-adjusted across the three group contrasts within each SAM outcome. Bold values indicate statistical significance after Holm correction (p_Holm < 0.05). SAM = Self-Assessment Manikin; HC = healthy control; L-stroke = left-hemisphere stroke; R-stroke = right-hemisphere stroke; MoCA = Montreal Cognitive Assessment; HAMA = Hamilton Anxiety Rating Scale; HAMD = Hamilton Depression Rating Scale; CI = confidence interval.

**Table S9. Descriptive statistics and statistical results for Power Spectral Density (PSD)**

| **Emotion** | **Healthy** | **Left Stroke** | **Right Stroke** | **Main Effect: Group** | **Main Effect: Emotion** | **Interaction Group×Emotion** |
| --- | --- | --- | --- | --- | --- | --- |
| **Theta Band: Frontal** | | | | | | |
| Negative | -1.836±2.853 | 0.832±2.782 | 0.074±2.939 |  |  |  |
| Neutral | -1.455±2.756 | 1.315±2.351 | 1.043±3.005 | Wald χ²(2) = 6.951, p = 0.031, p_FDR = 0.053 | **Wald χ²(2) = 18.023, p < 0.001, p_FDR < 0.001** | Wald χ²(4) = 5.140, p = 0.273, p_FDR = 0.298 |
| Positive | -1.680±3.478 | 0.593±3.095 | 0.351±2.686 |  |  |  |
| **Theta Band: Parietal** | | | | | | |
| Negative | -2.385±3.049 | 0.327±2.872 | -0.696±2.755 |  |  |  |
| Neutral | -1.739±2.861 | 0.604±2.781 | 0.008±2.426 | **Wald χ²(2) = 8.815, p = 0.012, p_FDR = 0.027** | **Wald χ²(2) = 17.041, p < 0.001, p_FDR < 0.001** | **Wald χ²(4) = 12.579, p = 0.014, p_FDR = 0.027** |
| Positive | -2.307±3.531 | 0.585±2.811 | -0.434±2.649 |  |  |  |
| **Alpha Band: Frontal** | | | | | | |
| Negative | -4.564±3.231 | -1.956±2.724 | -1.734±3.647 |  |  |  |
| Neutral | -3.835±3.073 | -1.355±2.716 | -0.792±3.458 | Wald χ²(2) = 4.247, p = 0.120, p_FDR = 0.160 | **Wald χ²(2) = 21.191, p < 0.001, p_FDR < 0.001** | Wald χ²(4) = 2.917, p = 0.572, p_FDR = 0.572 |
| Positive | -4.003±3.564 | -1.621±2.833 | -1.517±3.509 |  |  |  |
| **Alpha Band: Parietal** | | | | | | |
| Negative | -4.136±3.225 | -1.680±2.667 | -1.670±3.668 |  |  |  |
| Neutral | -3.682±3.261 | -1.241±2.533 | -0.871±3.274 | Wald χ²(2) = 6.138, p = 0.046, p_FDR = 0.070 | **Wald χ²(2) = 22.334, p < 0.001, p_FDR < 0.001** | Wald χ²(4) = 6.323, p = 0.176, p_FDR = 0.212 |
| Positive | -3.851±3.261 | -1.464±2.759 | -1.662±3.578 |  |  |  |

**Note:** Values represent mean band power ± standard deviation (dB/Hz). Bold values indicate statistical significance (p_FDR < 0.05).

**Table S10. Descriptive statistics and statistical results for emotion-averaged Lateralization Index (LI)**

| **Emotion** | **Healthy** | **Left Stroke** | **Right Stroke** | **Main Effect: Group** | **Main Effect: Emotion** | **Interaction Group×Emotion** |
| --- | --- | --- | --- | --- | --- | --- |
| **Theta Band: Frontal** | | | | | | |
| Negative | -0.001±0.116 | 0.049±0.204 | -0.160±0.152 |  |  |  |
| Neutral | -0.004±0.148 | 0.021±0.168 | -0.137±0.169 | Wald χ²(2) = 8.283, p = 0.016, p_FDR = 0.064 | Wald χ²(2) = 0.769, p = 0.681, p_FDR = 0.817 | Wald χ²(4) = 3.799, p = 0.434, p_FDR = 0.817 |
| Positive | -0.023±0.114 | 0.041±0.195 | -0.122±0.131 |  |  |  |
| **Theta Band: Parietal** | | | | | | |
| Negative | 0.015±0.112 | 0.255±0.189 | -0.030±0.205 |  |  |  |
| Neutral | 0.006±0.119 | 0.226±0.188 | -0.011±0.206 | **Wald χ²(2) = 17.638, p < 0.001, p_FDR < 0.001** | Wald χ²(2) = 0.140, p = 0.932, p_FDR = 0.932 | Wald χ²(4) = 3.116, p = 0.539, p_FDR = 0.817 |
| Positive | 0.015±0.153 | 0.225±0.185 | -0.060±0.156 |  |  |  |
| **Alpha Band: Frontal** | | | | | | |
| Negative | 0.003±0.128 | 0.001±0.170 | -0.074±0.127 |  |  |  |
| Neutral | 0.012±0.129 | -0.025±0.127 | -0.062±0.118 | Wald χ²(2) = 2.265, p = 0.322, p_FDR = 0.817 | Wald χ²(2) = 0.881, p = 0.644, p_FDR = 0.817 | Wald χ²(4) = 2.600, p = 0.627, p_FDR = 0.817 |
| Positive | -0.013±0.122 | -0.002±0.156 | -0.051±0.111 |  |  |  |
| **Alpha Band: Parietal** | | | | | | |
| Negative | 0.035±0.091 | 0.221±0.176 | -0.055±0.178 |  |  |  |
| Neutral | 0.027±0.096 | 0.190±0.176 | -0.065±0.179 | **Wald χ²(2) = 21.504, p < 0.001, p_FDR < 0.001** | Wald χ²(2) = 0.497, p = 0.780, p_FDR = 0.851 | Wald χ²(4) = 2.464, p = 0.651, p_FDR = 0.817 |
| Positive | 0.047±0.109 | 0.204±0.179 | -0.097±0.133 |  |  |  |

**Note:** LI was calculated as (Left − Right) / (Left + Right), where positive values indicate a leftward bias and negative values indicate a rightward bias. Values represent mean LI ± standard deviation. Bold values indicate statistical significance (p_FDR < 0.05).

**Table S11. Model-based effect estimates and 95% confidence intervals for primary within-clip parietal-theta LI dynamic outcomes at τ = 0.10**

| **Indicator** | **Simple-effect family** | **Stratum** | **Contrast** | **Estimate scale** | **Estimate** | **95% CI** |
| --- | --- | --- | --- | --- | --- | --- |
| R_det_win_ | Group within emotion | Neutral | HC vs L-stroke | OR | 0.383 | [0.218, 0.673] |
| R_det_win_ | Group within emotion | Neutral | HC vs R-stroke | OR | 0.685 | [0.387, 1.212] |
| R_det_win_ | Group within emotion | Neutral | L-stroke vs R-stroke | OR | 1.790 | [1.006, 3.182] |
| R_det_win_ | Group within emotion | Negative | HC vs L-stroke | OR | 0.381 | [0.217, 0.669] |
| R_det_win_ | Group within emotion | Negative | HC vs R-stroke | OR | 0.719 | [0.406, 1.272] |
| R_det_win_ | Group within emotion | Negative | L-stroke vs R-stroke | OR | 1.889 | [1.064, 3.357] |
| R_det_win_ | Group within emotion | Positive | HC vs L-stroke | OR | 0.466 | [0.265, 0.818] |
| R_det_win_ | Group within emotion | Positive | HC vs R-stroke | OR | 0.938 | [0.531, 1.658] |
| R_det_win_ | Group within emotion | Positive | L-stroke vs R-stroke | OR | 2.013 | [1.134, 3.571] |
| R_det_win_ | Emotion within group | HC | Neutral vs Negative | OR | 1.065 | [0.960, 1.183] |
| R_det_win_ | Emotion within group | HC | Neutral vs Positive | OR | 0.886 | [0.798, 0.983] |
| R_det_win_ | Emotion within group | HC | Negative vs Positive | OR | 0.831 | [0.750, 0.922] |
| R_det_win_ | Emotion within group | L-stroke | Neutral vs Negative | OR | 1.060 | [0.930, 1.207] |
| R_det_win_ | Emotion within group | L-stroke | Neutral vs Positive | OR | 1.078 | [0.949, 1.226] |
| R_det_win_ | Emotion within group | L-stroke | Negative vs Positive | OR | 1.018 | [0.897, 1.154] |
| R_det_win_ | Emotion within group | R-stroke | Neutral vs Negative | OR | 1.119 | [0.989, 1.266] |
| R_det_win_ | Emotion within group | R-stroke | Neutral vs Positive | OR | 1.213 | [1.076, 1.367] |
| R_det_win_ | Emotion within group | R-stroke | Negative vs Positive | OR | 1.084 | [0.964, 1.219] |
| SwitchRate | Group within emotion | Neutral | HC vs L-stroke | OR | 4.197 | [1.905, 9.246] |
| SwitchRate | Group within emotion | Neutral | HC vs R-stroke | OR | 1.809 | [0.813, 4.024] |
| SwitchRate | Group within emotion | Neutral | L-stroke vs R-stroke | OR | 0.431 | [0.193, 0.964] |
| SwitchRate | Group within emotion | Negative | HC vs L-stroke | OR | 4.654 | [2.112, 10.258] |
| SwitchRate | Group within emotion | Negative | HC vs R-stroke | OR | 1.720 | [0.773, 3.827] |
| SwitchRate | Group within emotion | Negative | L-stroke vs R-stroke | OR | 0.370 | [0.165, 0.827] |
| SwitchRate | Group within emotion | Positive | HC vs L-stroke | OR | 3.291 | [1.496, 7.239] |
| SwitchRate | Group within emotion | Positive | HC vs R-stroke | OR | 1.169 | [0.526, 2.597] |
| SwitchRate | Group within emotion | Positive | L-stroke vs R-stroke | OR | 0.355 | [0.159, 0.793] |
| SwitchRate | Emotion within group | HC | Neutral vs Negative | OR | 1.038 | [0.899, 1.199] |
| SwitchRate | Emotion within group | HC | Neutral vs Positive | OR | 1.122 | [0.973, 1.293] |
| SwitchRate | Emotion within group | HC | Negative vs Positive | OR | 1.080 | [0.937, 1.245] |
| SwitchRate | Emotion within group | L-stroke | Neutral vs Negative | OR | 1.152 | [0.965, 1.373] |
| SwitchRate | Emotion within group | L-stroke | Neutral vs Positive | OR | 0.879 | [0.742, 1.042] |
| SwitchRate | Emotion within group | L-stroke | Negative vs Positive | OR | 0.764 | [0.643, 0.907] |
| SwitchRate | Emotion within group | R-stroke | Neutral vs Negative | OR | 0.987 | [0.838, 1.163] |
| SwitchRate | Emotion within group | R-stroke | Neutral vs Positive | OR | 0.725 | [0.619, 0.849] |
| SwitchRate | Emotion within group | R-stroke | Negative vs Positive | OR | 0.734 | [0.627, 0.860] |
| Entropy | Group within emotion | Neutral | HC vs L-stroke | logit-scale difference | 2.258 | [0.721, 3.796] |
| Entropy | Group within emotion | Neutral | HC vs R-stroke | logit-scale difference | 1.317 | [-0.248, 2.882] |
| Entropy | Group within emotion | Neutral | L-stroke vs R-stroke | logit-scale difference | -0.942 | [-2.507, 0.623] |
| Entropy | Group within emotion | Negative | HC vs L-stroke | logit-scale difference | 2.478 | [0.941, 4.016] |
| Entropy | Group within emotion | Negative | HC vs R-stroke | logit-scale difference | 0.989 | [-0.576, 2.554] |
| Entropy | Group within emotion | Negative | L-stroke vs R-stroke | logit-scale difference | -1.489 | [-3.054, 0.076] |
| Entropy | Group within emotion | Positive | HC vs L-stroke | logit-scale difference | 1.861 | [0.323, 3.398] |
| Entropy | Group within emotion | Positive | HC vs R-stroke | logit-scale difference | 0.189 | [-1.376, 1.754] |
| Entropy | Group within emotion | Positive | L-stroke vs R-stroke | logit-scale difference | -1.672 | [-3.237, -0.107] |
| Entropy | Emotion within group | HC | Neutral vs Negative | logit-scale difference | 0.251 | [-0.305, 0.808] |
| Entropy | Emotion within group | HC | Neutral vs Positive | logit-scale difference | 0.437 | [-0.119, 0.994] |
| Entropy | Emotion within group | HC | Negative vs Positive | logit-scale difference | 0.186 | [-0.371, 0.743] |
| Entropy | Emotion within group | L-stroke | Neutral vs Negative | logit-scale difference | 0.471 | [-0.085, 1.028] |
| Entropy | Emotion within group | L-stroke | Neutral vs Positive | logit-scale difference | 0.040 | [-0.517, 0.596] |
| Entropy | Emotion within group | L-stroke | Negative vs Positive | logit-scale difference | -0.432 | [-0.988, 0.125] |
| Entropy | Emotion within group | R-stroke | Neutral vs Negative | logit-scale difference | -0.076 | [-0.651, 0.499] |
| Entropy | Emotion within group | R-stroke | Neutral vs Positive | logit-scale difference | -0.691 | [-1.265, -0.116] |
| Entropy | Emotion within group | R-stroke | Negative vs Positive | logit-scale difference | -0.615 | [-1.189, -0.040] |

**Note:** This table reports all planned post hoc simple-effect contrasts for the primary within-clip parietal-theta LI dynamic outcomes, rather than only statistically significant contrasts. R_det_win_ and SwitchRate were analyzed using binomial generalized linear mixed-effects models, and effect estimates are reported as odds ratios (ORs). For ORs, values < 1 indicate lower odds for the first level relative to the second level in the listed contrast; values > 1 indicate higher odds for the first level. Entropy estimates are reported on the model scale after boundary correction and logit transformation; positive values indicate higher logit-transformed entropy in the first level relative to the second level in the listed contrast. LI = lateralization index; R_det_win_ = within-clip detectability; SwitchRate = switching rate; CI = confidence interval; OR = odds ratio.

**Table S12. Sensitivity analysis of primary parietal-theta within-clip LI dynamics adjusting for baseline MoCA, HAMA, and HAMD scores**

| **Indicator** | **Main Effect: Group** | **Main Effect: Emotion** | **Interaction: Group×Emotion** | **Covariates (MoCA, HAMA, HAMD)** |
| --- | --- | --- | --- | --- |
| R_det_win_ | **Wald χ²(2) = 11.132, p = 0.004, p_FDR = 0.009** | Wald χ²(2) = 6.127, p = 0.047, p_FDR = 0.070 | **Wald χ²(4) = 32.757, p < 0.001, p_FDR < 0.001** | Wald χ²(3) = 1.091, p = 0.779, p_FDR = 0.779 |
| SwitchRate | **Wald χ²(2) = 13.129, p = 0.001, p_FDR = 0.004** | **Wald χ²(2) = 13.043, p = 0.001, p_FDR = 0.004** | **Wald χ²(4) = 49.262, p < 0.001, p_FDR < 0.001** | Wald χ²(3) = 2.775, p = 0.428, p_FDR = 0.513 |
| Entropy | **Wald χ²(2) = 10.474, p = 0.005, p_FDR = 0.009** | Wald χ²(2) = 5.126, p = 0.077, p_FDR = 0.103 | **Wald χ²(4) = 14.628, p = 0.006, p_FDR = 0.009** | Wald χ²(3) = 1.789, p = 0.617, p_FDR = 0.673 |

**Note:** Models included fixed effects for Group, Emotion, Group × Emotion, and baseline MoCA, HAMA, and HAMD scores. R_det_win_ and SwitchRate were analyzed using binomial generalized linear mixed-effects models, whereas Entropy was analyzed after boundary correction and logit transformation using a linear mixed-effects model. The covariate column represents the joint 3-df test of MoCA, HAMA, and HAMD. p_FDR values were Benjamini-Hochberg corrected within this covariate-adjusted EEG sensitivity-analysis family. Bold values indicate statistical significance (p_FDR < 0.05). LI = lateralization index; MoCA = Montreal Cognitive Assessment; HAMD = Hamilton Depression Rating Scale; HAMA = Hamilton Anxiety Rating Scale.

**Table S13.** **Brainstem-exclusion sensitivity analysis for behavioral ratings and primary parietal-theta within-clip LI dynamics**

| **Indicator** | **Main Effect: Group** | **Main Effect: Emotion** | **Interaction: Group × Emotion** |
| --- | --- | --- | --- |
| Valence | Wald χ²(2) = 5.042, p = 0.080, p_FDR = 0.080 | **Wald χ²(2) = 676.079, p < 0.001, p_FDR < 0.001** | **Wald χ²(4) = 16.273, p = 0.003, p_FDR = 0.004** |
| Arousal | **Wald χ²(2) = 24.138, p < 0.001, p_FDR < 0.001** | **Wald χ²(2) = 403.779, p < 0.001, p_FDR < 0.001** | **Wald χ²(4) = 15.643, p = 0.004, p_FDR = 0.005** |
| R_det_win_ | **Wald χ²(2) = 19.005, p < 0.001, p_FDR < 0.001** | Wald χ²(2) = 5.225, p = 0.073, p_FDR = 0.080 | **Wald χ²(4) = 32.680, p < 0.001, p_FDR < 0.001** |
| SwitchRate | **Wald χ²(2) = 21.589, p < 0.001, p_FDR < 0.001** | **Wald χ²(2) = 14.599, p < 0.001, p_FDR = 0.001** | **Wald χ²(4) = 52.089, p < 0.001, p_FDR < 0.001** |
| Entropy | **Wald χ²(2) = 17.073, p < 0.001, p_FDR < 0.001** | Wald χ²(2) = 5.085, p = 0.079, p_FDR = 0.080 | **Wald χ²(4) = 14.923, p = 0.005, p_FDR = 0.006** |

**Note:** Analyses were repeated after excluding the two patients with brainstem lesions. The original three-level Group factor was retained. R_det_win_ and SwitchRate were analyzed using binomial generalized linear mixed-effects models, whereas Entropy was analyzed after boundary correction and logit transformation using a linear mixed-effects model. Models included fixed effects for Group, Emotion, and Group × Emotion, with random intercepts for Subject and, for EEG dynamic LI outcomes, Clip. FDR correction was applied separately for each model effect across the outcomes in this sensitivity-analysis family. The Valence and Entropy models yielded singular-fit warnings; therefore, these results should be interpreted cautiously as sensitivity analyses. Bold values indicate statistical significance (p_FDR < 0.05). LI = lateralization index; R_det_win_ = within-clip detectability; SwitchRate = switching rate; p_FDR = false discovery rate-adjusted p value.

**Table S14.** **Exploratory stroke-only sensitivity analysis of primary parietal-theta within-clip LI dynamics after excluding brainstem lesions and adjusting for lesion category**

| **Outcome** | **Main Effect: Lesion Side** | **Main Effect: Emotion** | **Interaction: Lesion Side × Emotion** | **Lesion Category** |
| --- | --- | --- | --- | --- |
| R_det_win_ | **Wald χ²(1) = 7.196, p = 0.007, p_FDR = 0.025** | **Wald χ²(2) = 9.577, p = 0.008, p_FDR = 0.025** | Wald χ²(2) = 3.130, p = 0.209, p_FDR = 0.220 | Wald χ²(1) = 3.533, p = 0.060, p_FDR = 0.103 |
| SwitchRate | **Wald χ²(1) = 7.798, p = 0.005, p_FDR = 0.025** | **Wald χ²(2) = 45.160, p < 0.001, p_FDR < 0.001** | Wald χ²(2) = 4.731, p = 0.094, p_FDR = 0.126 | Wald χ²(1) = 2.008, p = 0.156, p_FDR = 0.188 |
| Entropy | **Wald χ²(1) = 5.643, p = 0.018, p_FDR = 0.035** | **Wald χ²(2) = 8.151, p = 0.017, p_FDR = 0.035** | Wald χ²(2) = 4.715, p = 0.095, p_FDR = 0.126 | Wald χ²(1) = 1.505, p = 0.220, p_FDR = 0.220 |

**Note:** Analyses were conducted in stroke patients only after excluding brainstem lesions. Lesion side was modeled as L-stroke versus R-stroke, and broad lesion category was modeled as cortical versus subcortical. R_det_win_ and SwitchRate were analyzed using binomial generalized linear mixed-effects models, whereas Entropy was analyzed after boundary correction and logit transformation using a linear mixed-effects model. Models included fixed effects for Lesion Side, Emotion, Lesion Side × Emotion, and Lesion Category, with random intercepts for Subject and Clip. FDR correction was applied separately for each model effect across the three primary dynamic LI outcomes. The Entropy model yielded a singular-fit warning; therefore, this exploratory sensitivity analysis should be interpreted cautiously. Bold values indicate statistical significance (p_FDR < 0.05). LI = lateralization index; R_det_win_ = within-clip detectability; SwitchRate = switching rate; p_FDR = false discovery rate-adjusted p value.

**Table S15.** **Sensitivity analysis of parietal-theta within-clip LI dynamics using data-driven thresholds**

| **Indicator** | **Main Effect: Group** | **Main Effect: Emotion** | **Interaction Group×Emotion** |
| --- | --- | --- | --- |
| **Sensitivity analysis at τ = 0.342 (95%)** | | | |
| R_det_win_ | **Wald χ²(2) = 17.612, p < 0.001, p_FDR < 0.001** | Wald χ²(2) = 5.024, p = 0.081, p_FDR = 0.184 | **Wald χ²(4) = 129.262, p < 0.001, p_FDR < 0.001** |
| SwitchRate | **Wald χ²(2) = 13.691, p = 0.001, p_FDR = 0.004** | **Wald χ²(2) = 9.032, p = 0.011, p_FDR = 0.033** | **Wald χ²(4) = 43.345, p < 0.001, p_FDR < 0.001** |
| Entropy | **Wald χ²(2) = 8.768, p = 0.012, p_FDR = 0.025** | Wald χ²(2) = 4.202, p = 0.122, p_FDR = 0.184 | **Wald χ²(4) = 15.323, p = 0.004, p_FDR = 0.007** |
| **Sensitivity analysis at τ = 0.273 (90%)** | | | |
| R_det_win_ | **Wald χ²(2) = 17.799, p < 0.001, p_FDR < 0.001** | **Wald χ²(2) = 7.831, p = 0.020, p_FDR = 0.027** | **Wald χ²(4) = 133.316, p < 0.001, p_FDR < 0.001** |
| SwitchRate | **Wald χ²(2) = 15.792, p < 0.001, p_FDR < 0.001** | **Wald χ²(2) = 8.468, p = 0.014, p_FDR = 0.022** | **Wald χ²(4) = 48.780, p < 0.001, p_FDR < 0.001** |
| Entropy | Wald χ²(2) = 3.448, p = 0.178, p_FDR = 0.214 | **Wald χ²(2) = 10.959, p = 0.004, p_FDR = 0.008** | Wald χ²(4) = 8.110, p = 0.088, p_FDR = 0.105 |
| **Sensitivity analysis at τ = 0.497 (99%)** | | | |
| R_det_win_ | **Wald χ²(2) = 17.831, p < 0.001, p_FDR < 0.001** | Wald χ²(2) = 5.422, p = 0.066, p_FDR = 0.146 | **Wald χ²(4) = 55.863, p < 0.001, p_FDR < 0.001** |
| SwitchRate | Wald χ²(2) = 1.587, p = 0.452, p_FDR = 0.497 | **Wald χ²(2) = 12.289, p = 0.002, p_FDR = 0.008** | **Wald χ²(4) = 14.156, p = 0.007, p_FDR = 0.015** |
| Entropy | **Wald χ²(2) = 16.379, p < 0.001, p_FDR < 0.001** | Wald χ²(2) = 0.049, p = 0.976, p_FDR = 0.976 | Wald χ²(4) = 9.547, p = 0.049, p_FDR = 0.089 |

**Note:** Analysis was performed using empirical thresholds defined as the 90th, 95th, and 99th percentiles of the null distribution (τ = 0.273, 0.342, 0.497). Bold values indicate statistical significance (p_FDR < 0.05).

**Table S16. Exploratory stroke-only correlations between HAMD/HAMA scores and primary parietal-theta dynamic LI metrics**

| **Emotion summary** | **Clinical scale** | **EEG metric** | **Spearman’s rho** | **95% CI** | **p** | **p_FDR** |
| --- | --- | --- | --- | --- | --- | --- |
| Positive | HAMD | R_det_win_ | -0.043 | [-0.399, 0.339] | 0.825 | 0.964 |
| Positive | HAMD | SwitchRate | -0.009 | [-0.395, 0.366] | 0.964 | 0.964 |
| Positive | HAMD | Entropy | 0.034 | [-0.356, 0.441] | 0.862 | 0.964 |
| Positive | HAMA | R_det_win_ | -0.057 | [-0.440, 0.357] | 0.770 | 0.964 |
| Positive | HAMA | SwitchRate | 0.223 | [-0.212, 0.601] | 0.245 | 0.964 |
| Positive | HAMA | Entropy | 0.199 | [-0.224, 0.601] | 0.301 | 0.964 |
| Emotion-averaged | HAMD | R_det_win_ | 0.107 | [-0.262, 0.472] | 0.582 | 0.964 |
| Emotion-averaged | HAMD | SwitchRate | -0.089 | [-0.489, 0.338] | 0.645 | 0.964 |
| Emotion-averaged | HAMD | Entropy | -0.025 | [-0.431, 0.380] | 0.896 | 0.964 |
| Emotion-averaged | HAMA | R_det_win_ | 0.047 | [-0.342, 0.442] | 0.807 | 0.964 |
| Emotion-averaged | HAMA | SwitchRate | 0.113 | [-0.298, 0.488] | 0.560 | 0.964 |
| Emotion-averaged | HAMA | Entropy | 0.063 | [-0.349, 0.437] | 0.746 | 0.964 |

**Note:** Analyses were conducted in stroke patients only. Clip-level parietal-theta dynamic LI metrics were aggregated to the participant level before correlation analyses to avoid pseudoreplication. Positive = positive clips only; Emotion-averaged = average across positive, neutral, and negative conditions. p_FDR values were Benjamini-Hochberg corrected across the affective-symptom correlation family shown in this table. HAMD = Hamilton Depression Rating Scale; HAMA = Hamilton Anxiety Rating Scale; LI = lateralization index; R_det_win_ = within-clip detectability; SwitchRate = switching rate; CI = confidence interval.

**Table S17. Robustness analyses of primary parietal-theta dynamic LI outcomes adjusting for retained epoch number**

| **Robustness model** | **Indicator** | **Group × Emotion effect** | **Retained epoch effect** | **Singular-fit warning** |
| --- | --- | --- | --- | --- |
| Adjusted for retained epochs | R_det_win_ | **Wald χ²(4) = 35.538, p < 0.001, p_FDR < 0.001** | **Wald χ²(1) = 7.644, p = 0.006** | No |
| Adjusted for retained epochs | SwitchRate | **Wald χ²(4) = 52.610, p < 0.001, p_FDR < 0.001** | **Wald χ²(1) = 7.304, p = 0.007** | No |
| Adjusted for retained epochs | Entropy | **Wald χ²(4) = 14.903, p = 0.005, p_FDR = 0.005** | Wald χ²(1) = 1.719, p = 0.190 | Yes |

**Note:** Models were adjusted for retained epoch number to evaluate whether the primary parietal-theta dynamic LI effects were influenced by unequal epoch retention after artifact rejection. Retained epoch number was z-standardized before model entry. R_det_win_ and SwitchRate were analyzed using binomial generalized linear mixed-effects models, whereas Entropy was analyzed after boundary correction and logit transformation using a linear mixed-effects model. p values for the Group × Emotion effects were FDR-corrected across the three retained-epoch-adjusted robustness models. The Entropy model yielded a singular-fit warning; therefore, this result should be interpreted cautiously. Bold values indicate statistical significance (p_FDR < 0.05). LI = lateralization index; R_det_win_ = within-clip detectability; SwitchRate = switching rate; p_FDR = false discovery rate-adjusted p value.

**Table S18. Secondary analyses of inter-clip LI stability and within-clip LI dynamics in other ROI × band combinations**

| **Band×ROI** | **Main Effect: Group** | **Main Effect: Emotion** | **Interaction Group×Emotion** |
| --- | --- | --- | --- |
| **Inter-Clip LI Stability** | | | |
| **Standard Deviation (SD_trial_)** | | | |
| Theta Band: Frontal | Wald χ²(2) = 4.525, p = 0.104, p_FDR = 0.104 | Wald χ²(2) = 2.120, p = 0.346, p_FDR = 0.520 | Wald χ²(4) = 2.287, p = 0.683, p_FDR = 0.722 |
| Alpha Band: Frontal | Wald χ²(2) = 6.705, p = 0.035, p_FDR = 0.052 | Wald χ²(2) = 1.113, p = 0.573, p_FDR = 0.573 | Wald χ²(4) = 2.076, p = 0.722, p_FDR = 0.722 |
| Alpha Band: Parietal | Wald χ²(2) = 7.752, p = 0.021, p_FDR = 0.052 | Wald χ²(2) = 2.623, p = 0.269, p_FDR = 0.520 | Wald χ²(4) = 5.989, p = 0.200, p_FDR = 0.600 |
| **Detectability (R_det_)** | | | |
| Theta Band: Frontal | **Wald χ²(2) = 9.567, p = 0.008, p_FDR = 0.013** | **Wald χ²(2) = 9.622, p = 0.008, p_FDR = 0.012** | Wald χ²(4) = 9.933, p = 0.042, p_FDR = 0.086 |
| Alpha Band: Frontal | Wald χ²(2) = 1.917, p = 0.383, p_FDR = 0.383 | **Wald χ²(2) = 35.042, p < 0.001, p_FDR < 0.001** | Wald χ²(4) = 6.404, p = 0.171, p_FDR = 0.171 |
| Alpha Band: Parietal | **Wald χ²(2) = 12.710, p = 0.002, p_FDR = 0.005** | Wald χ²(2) = 0.095, p = 0.953, p_FDR = 0.953 | Wald χ²(4) = 9.152, p = 0.057, p_FDR = 0.086 |
| **Directional consistency (C_dir_)** | | | |
| Theta Band: Frontal | **Wald χ²(2) = 10.589, p = 0.005, p_FDR = 0.008** | Wald χ²(2) = 3.139, p = 0.208, p_FDR = 0.312 | Wald χ²(4) = 2.620, p = 0.623, p_FDR = 0.775 |
| Alpha Band: Frontal | Wald χ²(2) = 2.346, p = 0.309, p_FDR = 0.309 | **Wald χ²(2) = 12.205, p = 0.002, p_FDR = 0.007** | Wald χ²(4) = 1.785, p = 0.775, p_FDR = 0.775 |
| Alpha Band: Parietal | **Wald χ²(2) = 14.517, p < 0.001, p_FDR = 0.002** | Wald χ²(2) = 0.031, p = 0.984, p_FDR = 0.984 | Wald χ²(4) = 4.818, p = 0.306, p_FDR = 0.775 |
| **Within-Clip LI Dynamics** | | | |
| **Entropy** |  |  |  |
| Theta Band: Frontal | Wald χ²(2) = 2.326, p = 0.312, p_FDR = 0.469 | **Wald χ²(2) = 7.894, p = 0.019, p_FDR = 0.029** | **Wald χ²(4) = 14.129, p = 0.007, p_FDR = 0.021** |
| Alpha Band: Frontal | Wald χ²(2) = 0.744, p = 0.689, p_FDR = 0.689 | **Wald χ²(2) = 15.544, p < 0.001, p_FDR = 0.001** | Wald χ²(4) = 6.966, p = 0.138, p_FDR = 0.207 |
| Alpha Band: Parietal | **Wald χ²(2) = 14.779, p < 0.001, p_FDR = 0.002** | Wald χ²(2) = 0.924, p = 0.630, p_FDR = 0.630 | Wald χ²(4) = 2.627, p = 0.622, p_FDR = 0.622 |
| **Detectability (R_det_win_)** |  |  |  |
| Theta Band: Frontal | **Wald χ²(2) = 7.919, p = 0.019, p_FDR = 0.029** | **Wald χ²(2) = 29.795, p < 0.001, p_FDR < 0.001** | **Wald χ²(4) = 46.942, p < 0.001, p_FDR < 0.001** |
| Alpha Band: Frontal | Wald χ²(2) = 4.504, p = 0.105, p_FDR = 0.105 | **Wald χ²(2) = 140.423, p < 0.001, p_FDR < 0.001** | **Wald χ²(4) = 26.877, p < 0.001, p_FDR < 0.001** |
| Alpha Band: Parietal | **Wald χ²(2) = 18.110, p < 0.001, p_FDR < 0.001** | Wald χ²(2) = 4.906, p = 0.086, p_FDR = 0.086 | **Wald χ²(4) = 43.306, p < 0.001, p_FDR < 0.001** |
| **SwitchRate** |  |  |  |
| Theta Band: Frontal | Wald χ²(2) = 5.116, p = 0.077, p_FDR = 0.116 | **Wald χ²(2) = 38.893, p < 0.001, p_FDR < 0.001** | **Wald χ²(4) = 51.055, p < 0.001, p_FDR < 0.001** |
| Alpha Band: Frontal | Wald χ²(2) = 0.005, p = 0.997, p_FDR = 0.997 | **Wald χ²(2) = 210.276, p < 0.001, p_FDR < 0.001** | **Wald χ²(4) = 13.474, p = 0.009, p_FDR = 0.009** |
| Alpha Band: Parietal | **Wald χ²(2) = 14.218, p < 0.001, p_FDR = 0.002** | Wald χ²(2) = 0.946, p = 0.623, p_FDR = 0.623 | **Wald χ²(4) = 40.720, p < 0.001, p_FDR < 0.001** |

**Note:** Omnibus mixed-effects tests for Frontal-Theta, Frontal-Alpha, and Parietal-Alpha features at τ = 0.10. Bold values indicate statistical significance (p_FDR < 0.05).

**Table S19.** **Type III Wald χ² tests for brain–behavior coupling models of SwitchRate**

| **Self-Assessment Manikin** | **Model** | **Effect** | **Test** |
| --- | --- | --- | --- |
| Arousal | Main analysis | Group | **Wald χ²(2) = 18.047, p < 0.001** |
| Arousal | Main analysis | Emotion | **Wald χ²(2) = 23.176, p < 0.001** |
| Arousal | Main analysis | Arousal | **Wald χ²(1) = 30.371, p < 0.001** |
| Arousal | Main analysis | Group × Emotion | **Wald χ²(4) = 49.351, p < 0.001** |
| Arousal | Main analysis | Group × Arousal | **Wald χ²(2) = 11.115, p = 0.004** |
| Arousal | Sensitivity analysis | Group | **Wald χ²(2) = 21.165, p < 0.001** |
| Arousal | Sensitivity analysis | Emotion | **Wald χ²(2) = 15.832, p < 0.001** |
| Arousal | Sensitivity analysis | Arousal | **Wald χ²(1) = 18.499, p < 0.001** |
| Arousal | Sensitivity analysis | Group × Emotion | **Wald χ²(4) = 25.589, p < 0.001** |
| Arousal | Sensitivity analysis | Group × Arousal | Wald χ²(2) = 2.913, p = 0.233 |
| Arousal | Sensitivity analysis | Emotion × Arousal | Wald χ²(2) = 2.252, p = 0.324 |
| Arousal | Sensitivity analysis | Group × Emotion × Arousal | **Wald χ²(4) = 12.883, p = 0.012** |
| Valence | Main analysis | Group | **Wald χ²(2) = 18.098, p < 0.001** |
| Valence | Main analysis | Emotion | **Wald χ²(2) = 15.610, p < 0.001** |
| Valence | Main analysis | Valence | Wald χ²(1) = 2.241, p = 0.134 |
| Valence | Main analysis | Group × Emotion | **Wald χ²(4) = 30.078, p < 0.001** |
| Valence | Main analysis | Group ×Valence | Wald χ²(2) = 0.162, p = 0.922 |
| Valence | Sensitivity analysis | Group | **Wald χ²(2) = 14.017, p < 0.001** |
| Valence | Sensitivity analysis | Emotion | **Wald χ²(2) = 22.192, p < 0.001** |
| Valence | Sensitivity analysis | Valence | Wald χ²(1) = 0.001, p = 0.978 |
| Valence | Sensitivity analysis | Group × Emotion | **Wald χ²(4) = 13.195, p = 0.010** |
| Valence | Sensitivity analysis | Group × Valence | **Wald χ²(2) = 6.974, p = 0.031** |
| Valence | Sensitivity analysis | Emotion × Valence | **Wald χ²(2) = 7.573, p = 0.023** |
| Valence | Sensitivity analysis | Group × Emotion × Valence | **Wald χ²(4) = 29.676, p < 0.001** |

Results are shown for models predicting parietal-theta SwitchRate from SAM arousal and valence ratings. Bold values indicate statistical significance (p < 0.05).
